# Transcriptomic analysis reveals biosynthesis genes and transcription factors related to leaf anthocyanin biosynthesis in *Aglaonema commutatum*

**DOI:** 10.1186/s12864-022-09107-1

**Published:** 2023-01-17

**Authors:** Ji Li, Kunlin Wu, Lin Li, Guohua Ma, Lin Fang, Songjun Zeng

**Affiliations:** 1 Key Laboratory of South China Agricultural Plant Molecular Analysis and Gene Improvement, South China Botanical Garden, Chinese Academy of Sciences, Guangzhou, 510650 China; 2 University of Chinese Academy of Sciences, Beijing, 100049 China; 3 Guangdong Provincial Key Laboratory of Applied Botany, South China Botanical Garden, Chinese Academy of Sciences, Guangzhou, 510650 China; 4 Center of Economic Botany, Core Botanical Gardens, Chinese Academy of Sciences, Guangzhou, 510650 China

**Keywords:** *Aglaonema commutatum* ‘red valentine’, Transcriptomics, Anthocyanin biosynthesis, R2R3-MYB, Transcriptional regulation

## Abstract

**Background:**

*Aglaonema commutatum* ‘Red Valentine’, as a foliage ornamental plant, is widely used for interior and exterior decoration because of its easy cultivation and management. However, reduced proportion of red foliage during large-scale production of *A. commutatum* seedlings is a frequent occurrence, which has considerable implications on the plant’s ornamental and market value. However, the molecular mechanisms underlying this phenomenon remain unclear.

**Results:**

To explore the molecular basis of the variation in leaf color of *A. commutatum* Red Valentine, we performed transcriptome sequencing with the Illumina platform using two different varieties of *A. commutatum*, namely Red Valentine and a green mutant, at three different stages of leaf development. We annotated 63,621 unigenes and 14,186 differentially expressed genes by pairwise comparison. Furthermore, we identified 26 anthocyanin biosynthesis structural genes. The transcript per million (TPM) values were significantly higher for Red Valentine than for the green mutant in all three developmental stages, consistent with the high anthocyanin content of Red Valentine leaves. We detected positive transcription factors that may be involved in the regulation of anthocyanin biosynthesis using BLAST and through correlation analysis. Downregulation of these transcription factors may downregulate the expression of anthocyanin genes. We obtained full-length cDNA of the anthocyanin biosynthesis and regulatory genes and constructed phylogenetic trees to ensure accuracy of the analysis.

**Conclusions:**

Our study provides insights into the molecular mechanisms underlying leaf variation in *A. commutatum* Red Valentine and may be used to facilitate the breeding of ornamental cultivars with high anthocyanin levels.

**Supplementary Information:**

The online version contains supplementary material available at 10.1186/s12864-022-09107-1.

## Background

Flower, fruit, and leaf colors of agricultural crops are important traits of commercial and ornamental value and are determined by natural plant pigments, including anthocyanins, betalains, chlorophylls, and carotenoids. Among them, anthocyanins are the major pigments that confer red, violet, or blue color [1]. In plants, anthocyanins play vital roles in seed dispersal and stress responses [2, 3]; in humans, they serve as antioxidants and protect against cardiovascular diseases, cancers, and several chronic diseases. Moreover, they are widely used in the food, medicine, and cosmetic industries [4–6].

Studies have shown that the anthocyanin biosynthesis pathway is a ubiquitous branch of the flavonoid biosynthesis pathway in plants. Genes regulating anthocyanin biosynthesis and transport have been identified in numerous plant species [7–9]. This pathway starts from the phenylpropanoid pathway, involving three major genes: *PAL* (phenylalanine ammonia-lyase), *C4H* (trans-cinnamate 4-monooxygenase), and *4CL* (4-coumarate-CoA ligase 2). The flavonoid biosynthesis pathway includes two classes of genes, namely early (*EBGs*) and late biosynthesis genes (*LBGs*) [10]. Chalcone synthase (CHS), chalcone isomerase (CHI), flavanone 3-hydroxylase (F3H), flavonoid 3-hydroxylase (F3’H), and flavonol synthase (FLS) participate in early biosynthesis. These genes regulate the biosynthesis of flavonols and other flavonoid compounds, whereas dihydroflavonol-4-reductase (DFR), anthocyanidin synthase (ANS), and UDP-glucose flavonoid 3-O-glucosyltransferase (UFGT) regulate the biosynthesis of anthocyanins. Subsequently, water-soluble anthocyanins are transported into the vacuole for stable storage [8].

Transcriptional regulation is important for regulating the expression profiles of anthocyanin biosynthesis genes in several plant species, and it is primarily regulated by three types of transcription factors (TFs): the R2R3-MYB gene family, bHLH TFs, and WD40 repeat proteins [11]. EBGs are regulated by R2R3-MYB genes, whereas LBGs are activated by a transcriptional activation complex consisting of MYB, bHLH, and WD40 proteins (MBW) [10, 12]. In *Arabidopsis thaliana*, PAP1 is a key R2R3-MYB TF that regulates anthocyanins, and its overexpression can significantly induce anthocyanin accumulation, resulting in dark purple leaves [7]. Other R2R3-MYB TFs (PAP2, MYB113, and MYB114), bHLH gene family members (*TT8*, *GL3*, and *EGL3*) and the WD40 gene (*TTG1*) have been identified as the key TFs involved in anthocyanin biosynthesis [13, 14]. Important TFs that regulate anthocyanin structural genes have also been found in some ornamental plant species, including in *Gerbera hybrida* (GMYB10 and GMYC1 [15];), *Chrysanthemum morifolium* (CmMYB6 and CmbHLH [16, 17];), ornamental kale (BoTTG1 and BoTT8 [14, 18];), *Lilium* spp. (LhMYB6, LhMYB12, and LhbHLH1 [19, 20];), and *Anthurium andraeanum* (AaMYB2 and AabHLH1) [21, 22].

The name *Aglaonema* is used for collectively referring to a group of perennial flowering plants belonging to the family Araceae. These plants are native to tropical Asian countries, including India, Thailand, Vietnam, the Philippines, Malaysia, and Indonesia. *Aglaonema commutatum* is a plant species widely used for interior decoration and as a courtyard ornamental plant because of its easy cultivation and management. Its leaves can have a variety of colors and beautiful stripes, making it increasingly popular in the market as an excellent foliage and fresh-cut ornamental plant [23]. At present, large-scale production of *A. commutatum* seedlings is mainly performed through tissue culture. However, during the tissue culture process, cases of reduced proportions of red foliage have been observed; in some cases, the foliage changed from red to green. This phenomenon has considerable implications for the ornamental and market value of this species. In recent years, studies on *A. commutatum* have mainly focused on tissue culture [24–27], stress resistance, breeding [27], and interspecies genetic relationships at the phenotypic and biochemical level. On the other hand, relatively fewer studies have examined the mechanisms underlying leaf color variations in *A. commutatum*. Moreover, little is known about the mechanisms regulating anthocyanin biosynthesis in *A. commutatum*.

Here, the effect of developmental stages on the leaf color of two varieties of *A. commutatum* (Red Valentine and a green mutant) was examined at the biochemical and genomic levels. The total anthocyanin and chlorophyll contents and anthocyanin monomer compositions of leaves of the two varieties were examined at three leaf developmental stages (S1–S3). Additionally, the TFs and structural genes regulating anthocyanin biosynthesis were identified using RNA-seq technology.

## Results

### Leaf color changes during different leaf development stages

**Fig. 1:**
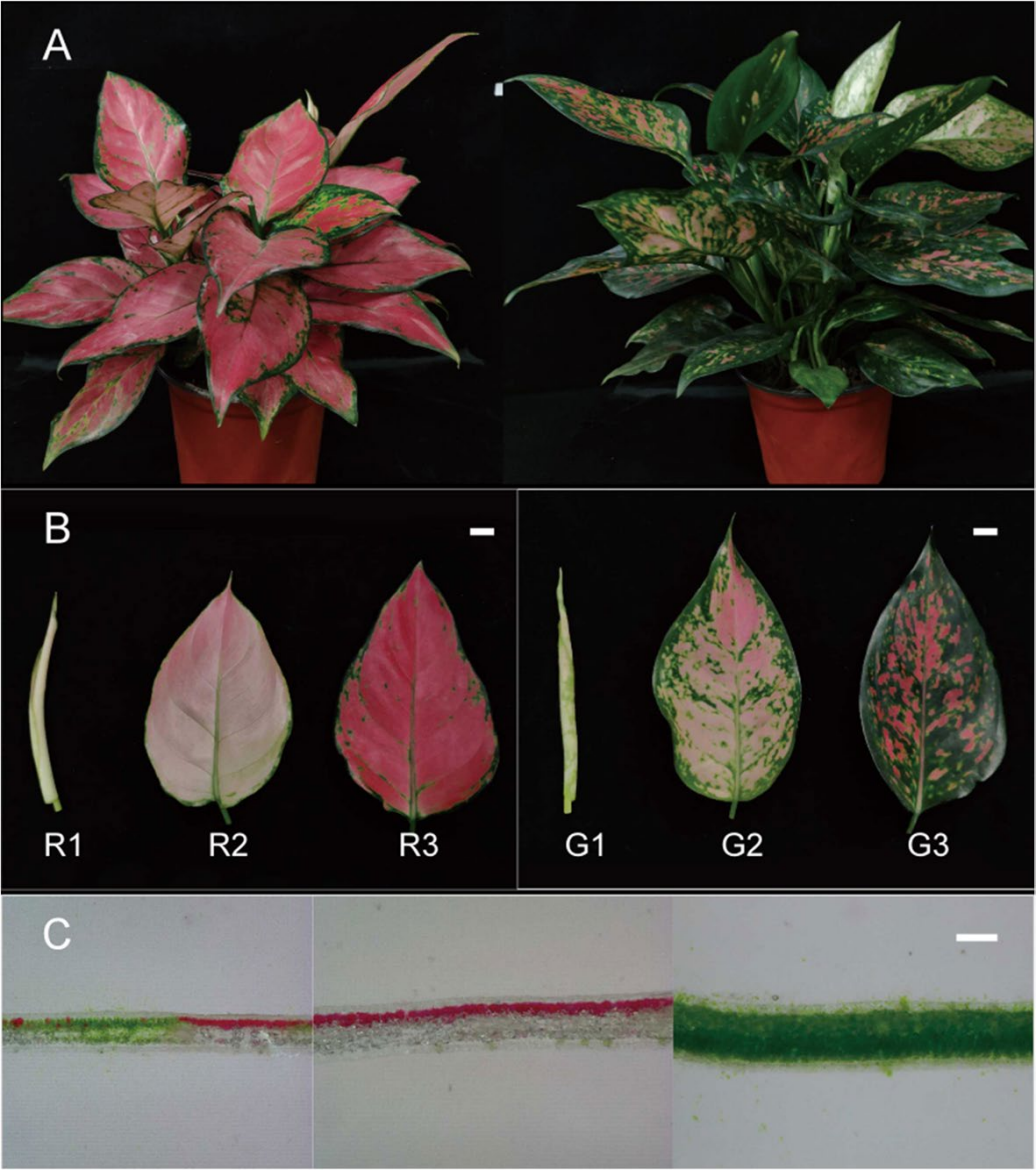
Morphological characteristics of *Aglaonema commutatum* leaves. **A** Pigmentation and phenotypes of *A. commutatum* Red Valentine and the green mutant. **B** Leaf phenotypes: stage 1, white curly leaves with small amount of pigment (7 d); stage 2, uncurled leaves exhibiting light pink coloration (28 d); stage 3: mature leaves exhibiting dark red coloration with oily surface (35 d). R1, R2, and R3: stages 1, 2, and 3 of *A. commutatum* Red Valentine, respectively; G1, G2, and G3: stages 1, 2 and 3 of the green mutant, respectively. The scale bars are 1 cm. **C** Anatomical observation of pigments in the lamina of *A. commutatum* Red Valentine and a green mutant. The scale bars are 100 μm

Except for the leaf color variation between the two *A. commutatum* varieties, no significant differences in the leaf shape and plant size were observed between the two varieties (Fig. 1A). Some changes in leaf appearance were observed during S1 to S2, namely leaf uncurling and color change from white to light pink and green. At maturity, the leaf color changed to deep red and dark green, and the leaf surface was shiny, indicating waxy coating. In the green mutant, red patches were concentrated in the middle of the leaves (Fig. 1B). To determine the distribution of pigments inside the leaf tissues, anatomical observations of leaf lamina of the two varieties at the three developmental stages were performed. Deep red pigmentation was observed only in the palisade tissues, and chlorophylls were observed in both the sponge and palisade tissues (Fig. 1C).

### Leaf chlorophyll and anthocyanin contents

**Fig. 2:**
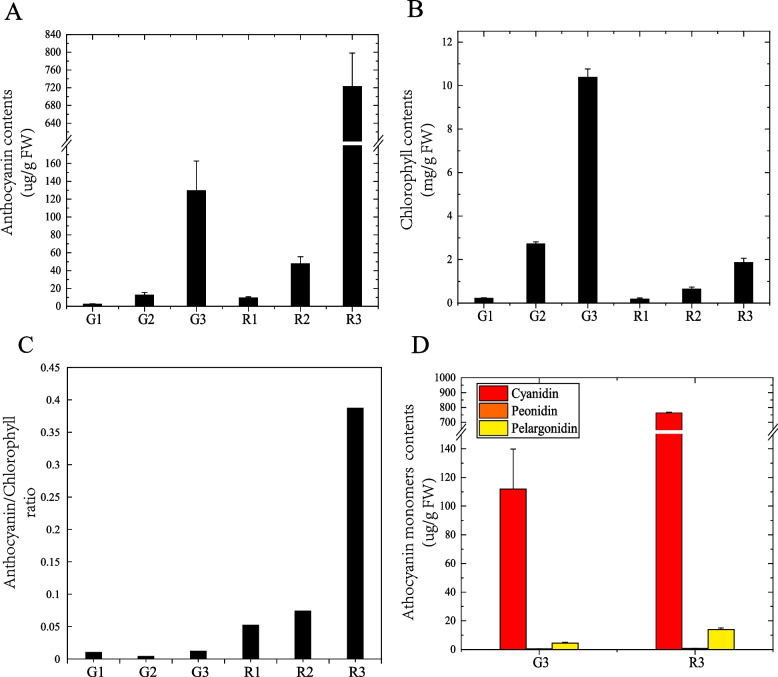
Pigment accumulation in the leaves of *Aglaonema commutatum* Red Valentine and a green mutant at three developmental stages. **A** Total anthocyanin content. **B** Total chlorophyll content. **C** Anthocyanin/chlorophyll ratio. **D** Anthocyanin monomer content

To check whether the color change was governed by the change in total anthocyanin and chlorophyll contents, the contents of the two pigments in the leaves at the three developmental stages were measured. A significant increase in both leaf anthocyanin and chlorophyll contents was observed in both varieties of *A. commutatum* from S1 to S2. However, the anthocyanin content of Red Valentine leaves was significantly higher than that of green mutant leaves in all three development stages. The leaf anthocyanin content of Red Valentine was approximately 5.58-fold higher than that of green mutant leaves at S3 (Fig. 2A). In contrast, the chlorophyll content of green mutant leaves was significantly higher than that of Red Valentine leaves in the three developmental stages. The chlorophyll content in green mutant leaves was approximately 5.57-fold higher than that in Red Valentine leaves at S3 (Fig. 2B). Furthermore, the leaf anthocyanin/chlorophyll ratio of the mutant at the three developmental stages was not significantly affected by the developmental stages, with the highest value (0.01) observed at S3. However, in Red Valentine, a significant increase in leaf anthocyanin/chlorophyll ratio was observed from S2 (0.07) to S3 (0.39), indicating that the mutants did not accumulate anthocyanins as much as the Red Valentine did (Fig. 2C).

Furthermore, HPLC was used to determine the composition of anthocyanin monomers in the two varieties at S3. Three anthocyanin monomers, including cyanidin, paeonidin, and pelargonin, were identified in the leaves. Among them, cyanidin was the most abundant, accounting for 98.1 and 95.8% pigments in the leaves of Red Valentine and the mutant, respectively, indicating that it was responsible for the red color of the leaves. Trace amounts of paeonidin and pelargonin were also detected (Fig. 2D). Our data implied that the composition and proportion of anthocyanin monomers contributed to the color changes in mature leaves, as no significant difference in the ratio of the two monomers was observed between the two varieties.

### Library construction and de novo assembly

A total of 18 high quality RNA samples were extracted from the leaves of the two *A. commutatum* varieties at the three developmental stages for further transcriptome analysis using Illumina sequencing with the HiSeq 6000 sequencer. The raw sequencing data were checked for quality and subjected to data filtering. The number of clean reads for each library ranged from 22,791,579 to 28,919,060, with a mapped ratio between 66.71 and 76.25%. The GC content ranged from 50.54 to 54.95%, and the Q30 base percentage was ≥94.71%. A total of 63,621 unigenes with an N50 length of 1340 bp were generated (Table 1).

**Table 1:** A summary of the sequencing data

Samples	Clean reads	Mapped reads	Mapped ratio(%)	Q30(≥%)	GC content(%)
G1_A	28,919,060	21,553,660	74.53	96.36	53.44
G1_B	26,875,623	20,492,131	76.25	96.37	53.33
G1_C	26,320,908	18,925,032	71.90	96.12	52.57
G2_A	22,817,364	16,390,186	71.83	95.62	53.34
G2_B	27,104,121	19,606,183	72.34	96.27	54.33
G2_C	23,676,524	16,923,445	71.48	95.86	53.4
G3_A	23,320,739	16,022,526	68.71	96.16	53.38
G3_B	25,671,029	17,124,158	66.71	95.97	52.65
G3_C	24,603,689	17,458,253	70.96	96.35	53.07
R1_A	23,431,089	17,426,287	74.37	96.24	54.63
R1_B	27,039,584	20,294,292	75.05	96.5	54.95
R1_C	23,244,555	16,316,706	70.20	95.67	53.9
R2_A	24,510,857	17,071,903	69.65	95.87	54.26
R2_B	24,249,840	17,898,965	73.81	96.39	54.05
R2_C	27,739,382	20,209,775	72.86	96.31	54.07
R3_A	22,791,579	15,490,014	67.96	94.71	50.54
R3_B	23,917,425	16,698,185	69.82	96.27	51.86
R3_C	23,008,761	16,564,656	71.99	96.49	53.21

### Functional annotation and classification

**Fig. 3:**
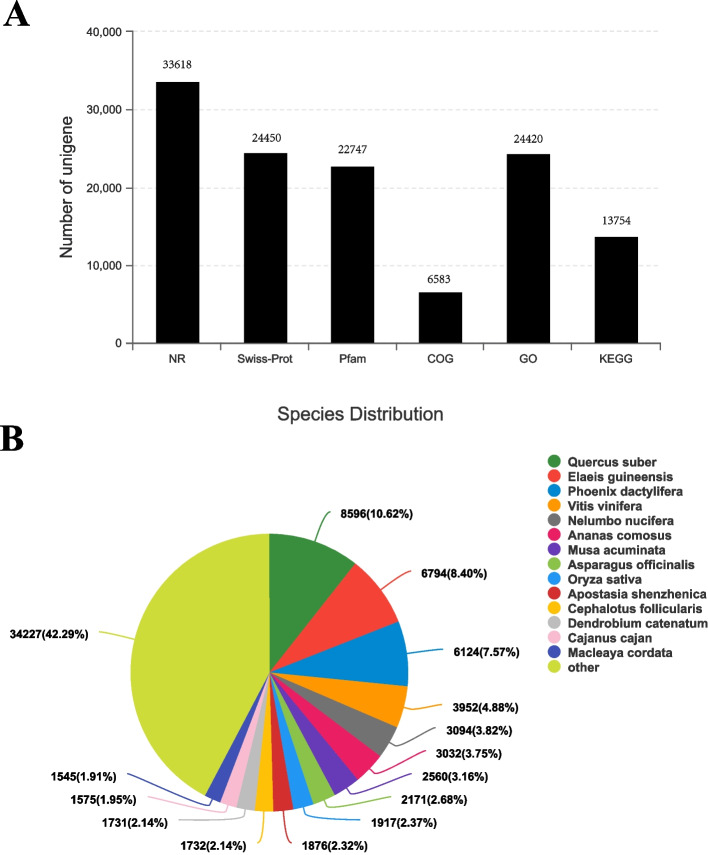
Functional annotation and classification. **A** Gene ontology (GO) classification of unigenes. **B** Kyoto Encyclopedia of Genes and Genomes (KEGG) pathway classification. **C** Numbers of functional annotation of all unigenes in six databases. **D** Species distribution of unigenes matching the main species obtained using BLASTx in the NR database

A total of 63,621 assembled unigenes were BLAST-searched against the NCBI non-redundant protein (NR), Nt, SwissProt, Kyoto Encyclopedia of Genes and Genomes (KEGG), KOG, Pfam, and Gene Ontology (GO) databases using BLASTx, and 34,554 unigenes were annotated (Fig. 3A). The unigenes were annotated and aligned in the NR database. *Quercus suber* had the highest matching degree (10.62%), followed by *Elaeis guineensis* (8.40%) and *Phoenix dactylifera* (7.57%). Approximately 42.29% of the unigene sequence did not match the protein sequences of the other species (Fig. 3B). Results of the GO analysis showed that the genes were mainly involved in metabolic processes, including cell part and compound binding, which were the major metabolic processes. Results of the KEGG analysis showed that the unigenes were involved in translation, carbohydrate metabolism, and folding, sorting, and degradation pathways (Supplementary Fig. S2).

### Identification and KEGG classification of differentially expressed genes (DEGs)

**Fig. 4:**
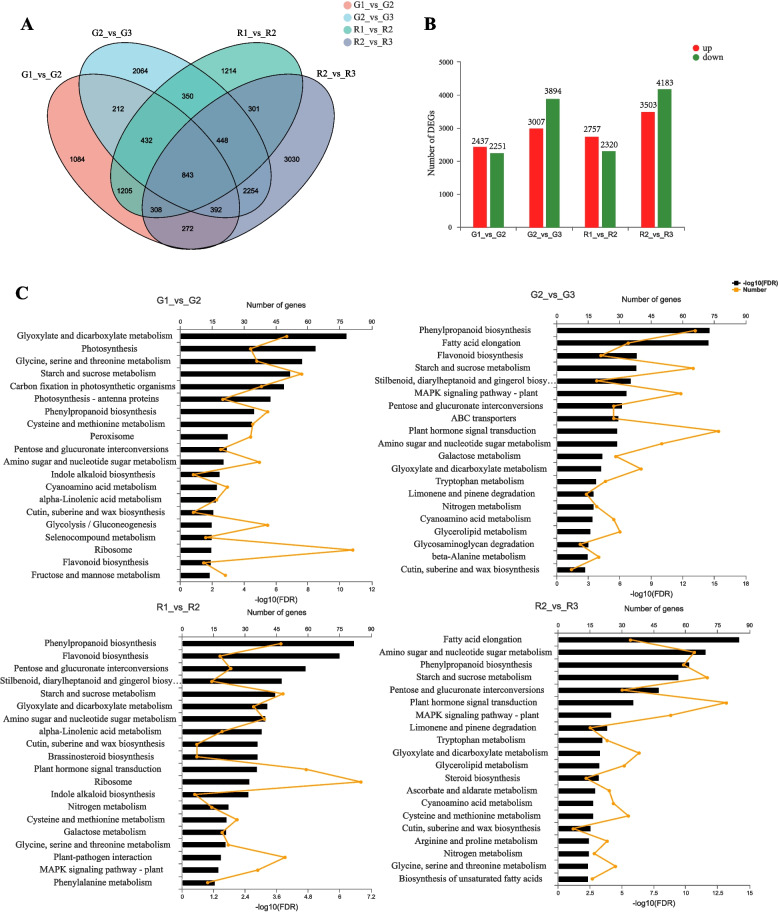
Identification of differentially expressed genes (DEGs) in *Aglaonema commutatum* Red Valentine. **A** Venn diagrams of the DEGs. **B** List of DEGs between pairs of treatments. **C** KEGG pathway enrichment of DEGs in G1 vs. G2, G2 vs. G3, R1 vs. R2, and R2 vs. R3

The transcript per million (TPM) values of the unigenes were used to identify DEGs between the leaves of Red Valentine and the green mutant at the three developmental stages using the DEGseq R package. A total of 14,186 DEGs (*p*-adjust ≤0.05; FC ≥ 2) were identified by pairwise comparison (G1 vs. G2, G2 vs. G3, R1 vs. R2, and R2 vs. R3). A Venn diagram of the DEGs showed that 843 DEGs were common to the four comparison groups (Fig. 4A). Among the four comparisons, the number of DEGs in S2 vs. S3 was higher than that in S1 vs. S2. The changes in gene expression are shown in Fig. 4B. Additionally, the KEGG pathway enrichment analysis showed that the DEGs were significantly enriched in glyoxylate and dicarboxylate metabolism, photosynthesis, phenylpropanoid biosynthesis, fatty acid elongation, flavonoid biosynthesis, and amino sugar and nucleotide sugar metabolism (Fig. 4C).

### Expression patterns of genes involved in anthocyanin biosynthesis

**Fig. 5:**
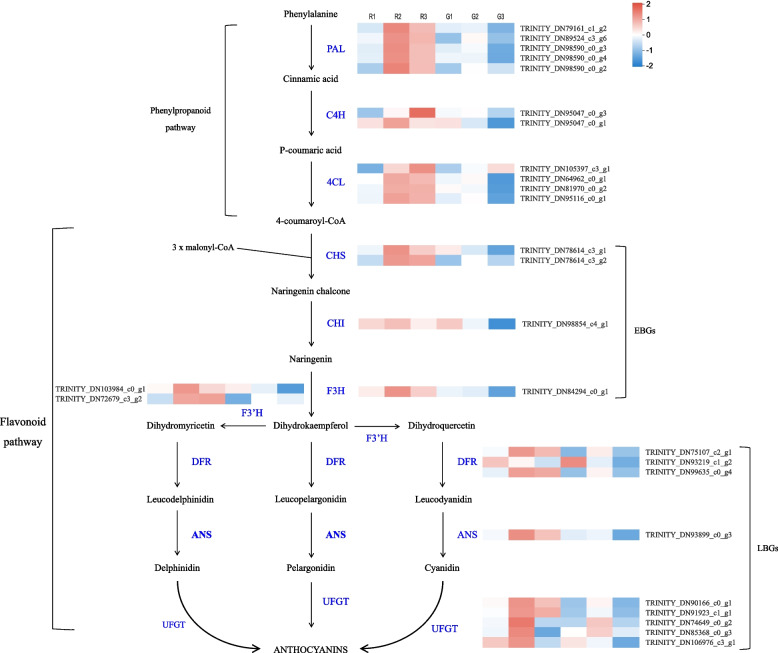
Heatmap of differentially expressed genes (DEGs) related to anthocyanin biosynthesis. The pathway can be divided into two sections: the phenylpropanoid and flavonoid pathways and early (EBGs) and late biosynthesis genes (LBGs) are involved in the flavonoid pathway

The expression pattern of the unigenes was determined using the TPM values. The expression profiles of the genes encoding enzymes related to anthocyanin biosynthesis in Red Valentine leaves showed that the phenylpropanoid pathway was regulated by three groups of genes, including five *PAL* unigenes, two *C4H*, and *4CL* unigenes. Two CHS, one CHI, and one F3H unigenes were expressed in the early biosynthesis stage. Accordingly, three DFR, one ANS, and five UFGTs unigenes were expressed during the later biosynthesis stage. Consistent with the high anthocyanin content of Red Valentine leaves, the TPM values of the anthocyanin structural unigenes were higher in the Red Valentine leaves than in the leaves of the green mutant at all three developmental stages. From stage R1 to stage R2, a significant increase (10-fold) in the expression profiles of most anthocyanin related genes (22 out of 26) was observed. Although the fold change was slightly lower than that observed from stage R1 to R2, the gene expression profiles also exhibited an upward trend from stage R2 to stage R3. Contrarily, a significant decrease in the gene expression profiles was observed in the leaves of the green mutant, with extremely low TPM values at all three developmental stages (Fig. 5). Full-length cDNA of the genes was obtained from RNA-seq data, and rapid amplification of cDNA ends (RACE), PCR, and phylogenetic tree construction were performed to ensure the accuracy of the analysis.

### Identification of TFs related to anthocyanin biosynthesis

**Fig. 6:**
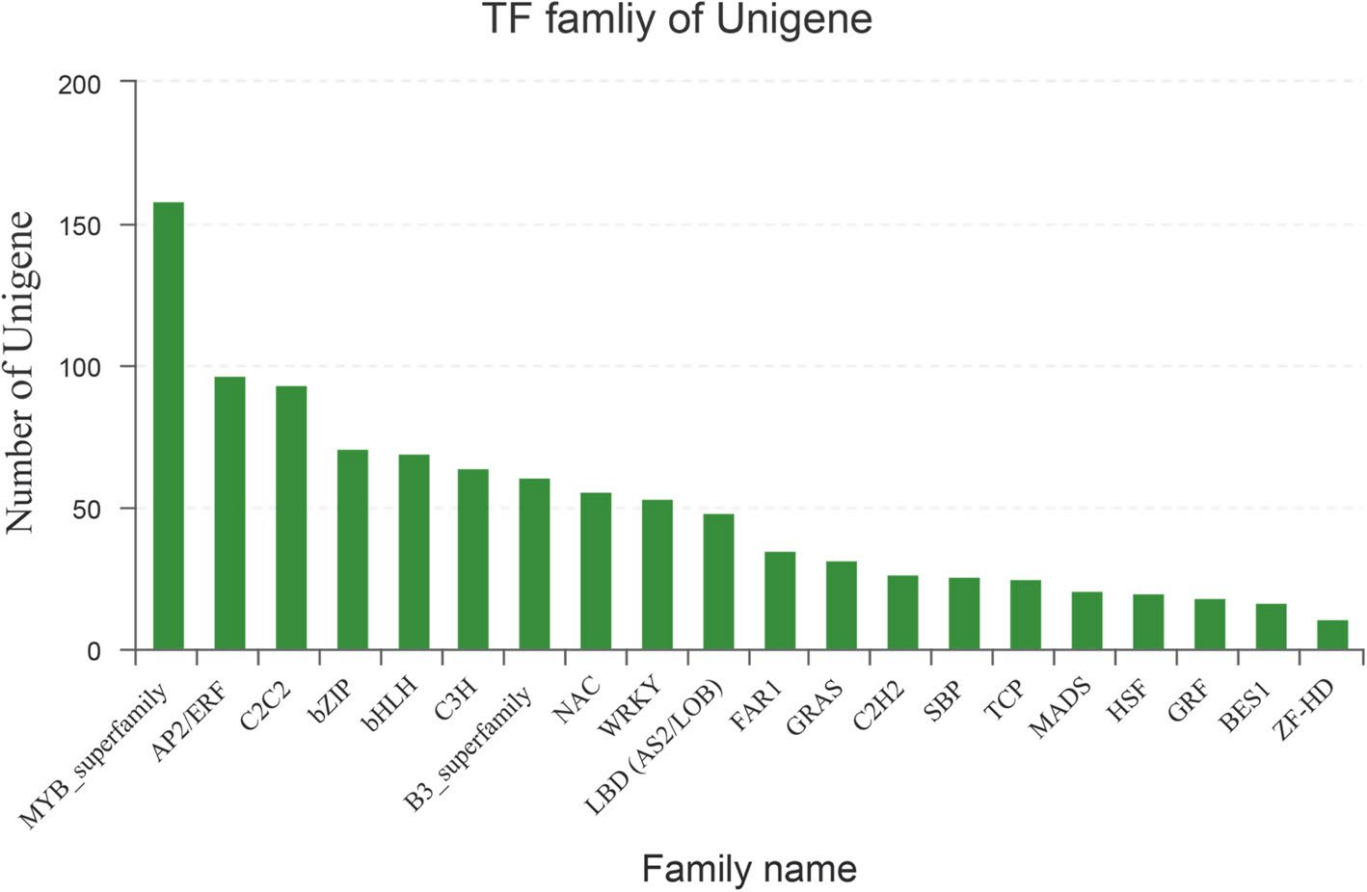
Top 20 transcription factors in the leaves of *Aglaonema commutatum* Red Valentine and a green mutant

Anthocyanin biosynthesis genes are mainly regulated by three different families of TFs, namely MYB, bHLH, and WDR. Thus, the TFs regulating anthocyanin biosynthesis in the leaves of *A. commutatum* were examined. A total of 1068 TFs belonging to 32 TF families were identified by aligning to the PlantTFDB database using BLASTX (Fig. 6). The top 10 annotated TFs included the MYB_superfamily, AP2/ERF, C2C2, bZIP, bHLH, C3H, B3_superfamily, NAC, WRKY, and LBD (AS2/LOB), each of which comprised over 48 unigenes in both varieties of *A. commutatum*. To identify MYB and WDR TFs related to anthocyanin biosynthesis, a functional search in the RNA-seq annotation file was performed, and the following unigene information was obtained. TRINITY_DN91028_c1_g1 (Unigene ID) was annotated to be a putative flavonoid or anthocyanin regulator (*A. andraeanum*). The gene *AaMYB1* in combination with bHLH genes has been reported to be associated with anthocyanin biosynthesis through biolistic transformation in a *Cymbidium* orchid [28]. Furthermore, TRINITY_DN62889_c2_g2 was annotated to have the highest homology with AaMYB2 (*A. andraeanum*), which was proven to primarily contribute to the regulation of anthocyanin biosynthesis in the spathes and leaves of *A. andraeanum* [22]. The results of qRT-PCR confirmed the profile of these two unigenes in relation to anthocyanin biosynthesis in *A. commutatum*. Moreover, TRINITY_DN84708_c1_g1 had a high similarity with TTG1, which is involved in anthocyanin biosynthesis regulation in many plant species [29, 30].

Regarding the bHLH TFs, we were unable to identify the bHLH unigenes involved in anthocyanin biosynthesis in the two varieties of *A. commutatum* based on the RNA-seq annotation data. Therefore, based on the expression profiles of the anthocyanin biosynthesis genes, we searched based on the expression profiles of anthocyanin biosynthesis genes. Red Valentine 26 putative anthocyanin biosynthesis genes (Date A) (Supplementary Table S1) and all bHLH TFs (Date B) were first normalized to reduce the error caused by exact TPM means. A correlation analysis was performed between the mean values of Date A (which could represent the anthocyanin biosynthesis gene expression level as a whole) and Date B to determine the relationship between each bHLH TF and the expression profiles of the anthocyanin biosynthesis genes, which were ranked in descending order (Table 2). The top 10 bHLH unigenes were aligned against the NCBI database (https://blast.ncbi.nlm.nih.gov/Blast.cgi) using BLASTX to obtain detailed information of the unigenes. We found that TRINITY_DN107490_c2_g1, TRINITY_DN8 7484_c1_g1, and TRINITY_DN97087_c0_g10 had the highest correlation coefficients with the basic helix-loop-helix TF 1 (*A. andraeanum*) annotation. *AabHLH1* was reported to participate in the regulation of the flavonoid metabolic pathway [21], suggesting that it may also be involved in the regulation of anthocyanin biosynthesis in Red Valentine*.* This gene was cloned using RACE PCR, and we found that the three unigenes belong to one gene named *AcbHLH1*. qRT-PCR revealed the gene expression patterns of *AcbHLH1*, which were consistent with the anthocyanin structural genes in the leaves of the two *A. commutatum* varieties. The CDS sequence of these TFs was cloned by sequence information and RACE PCR (Supplementary Table S2) for the phylogenetic tree analysis (Supplementary Fig. S3) and further studies, and they were named *AcMYB1*, *AcMYB2*, *AcTTG1* and *AcbHLH1*.

**Table 2:** Correlation coefficient between the mean value of anthocyanin biosynthetic genes expression level and bHLH TFs, arranged in descending order of correlation coefficient

Unigene ID	Correlation coefficent	NR_description from Transcriptomic date	Description from NCBI by blastx	Percent identity of blastx	Protein sequence ID
TRINITY_DN107490_c2_g1	0.9984	basic helix-loop-helix protein A [*Jatropha curcas*]	basic helix-loop-helix transcription factor 1, partial [*Anthurium andraeanum*]	0.7339	AZS49191.1
TRINITY_DN85407_c0_g2	0.9347	hypothetical protein CCACVL1_14859 [*Corchorus capsularis*]	basic helix-loop-helix transcription factor 1, partial [*Anthurium andraeanum*]	0.8793	AZS49191.1
TRINITY_DN87484_c1_g1	0.8675	PREDICTED: transcription factor bHLH47-like [*Phoenix dactylifera*]	transcription factor BHLH062-like [*Phoenix dactylifera*]	0.6905	XP_008807714.1
TRINITY_DN97087_c0_g10	0.8166	None(Pfam description:bHLH-MYC and R2R3-MYB transcription factors N-terminal)	basic helix-loop-helix transcription factor 1, partial [*Anthurium andraeanum*]	0.8462	AZS49191.1
TRINITY_DN89881_c0_g2	0.8144	PREDICTED: transcription factor HBI1 [*Nelumbo nucifera*]	PREDICTED: transcription factor HBI1 [*Nelumbo nucifera*]	0.4689	XP_010263637.1
TRINITY_DN87339_c0_g1	0.8140	PREDICTED: transcription factor FAMA [*Musa acuminata* subsp. malaccensis]	PREDICTED: transcription factor FAMA [*Musa acuminata* subsp. *malaccensis*]	0.6165	XP_018677740.1
TRINITY_DN67453_c0_g1	0.8058	PREDICTED: transcription factor UNE12-like isoform X1 [*Phoenix dactylifera*]	transcription factor UNE12 isoform X3 [*Elaeis guineensis*]	0.7469	XP_010936103.1
TRINITY_DN93501_c0_g1	0.7778	PREDICTED: transcription factor bHLH78-like isoform X1 [*Phoenix dactylifera*]	transcription factor bHLH62 [*Elaeis guineensis*]	0.4954	XP_029117287.1
TRINITY_DN100086_c1_g1	0.7504	PREDICTED: transcription factor bHLH13-like [*Phoenix dactylifera*]	transcription factor bHLH13-like [*Phoenix dactylifera*]	0.6493	XP_008806486.1
TRINITY_DN103801_c1_g1	0.7478	PREDICTED: transcription factor bHLH49 isoform X1 [*Elaeis guineensis*]	transcription factor bHLH49 isoform X1 [*Elaeis guineensis*]	0.5822	XP_010943352.1

### qRT-PCR analysis of anthocyanin-related DEGs

**Fig. 7:**
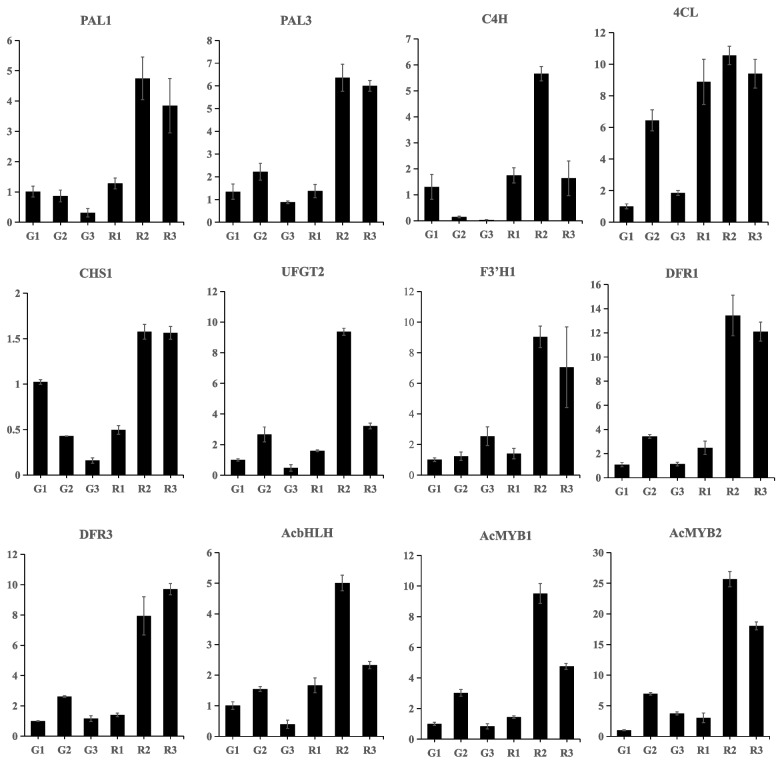
Comparative expression of candidate genes in the Red Valentine variety*.* Real-time qPCR of *PAL1* (TRINITY_DN98590_c0_g3); *PAL3* (TRINITY_DN89524_c3_g6); *C4H* (TRINITY_DN95047_c0_g1); *4CL* (TRINITY_DN95116_c0_g1); *CHS1* (TRINITY_DN78614_c3_g1); *UFGT2* (TRINITY_DN90166_c0_g1); *F3’H1* (TRINIY_DN103984_c0_g1); *DFR1* (TRINITY_DN75107_c2_g1); *DFR3* (TRINITY_DN99635_c0_g4); *AcbHLH*; *AcMYB1*; *AcMYB2* in *A. commutatum* Red Valentine leaves. The columns represent means ± standard deviations from three independent biological replicates

To validate the RNA-seq data, qRT-PCR assays were performed using gene-specific primers for the DEGs and TFs involved in anthocyanin biosynthesis. In total, nine unigenes of anthocyanin biosynthesis and identified TFs in the two varieties of *A. commutatum* were selected for qRT-PCR. The results of the qRT-PCR analysis generally showed expression patterns similar as the TPM values, indicating that the expression data obtained by RNA-seq were reliable (Fig. 7; Supplementary Table S3). Melting curves of the selected gene are shown in Supplementary Fig. S4.

## Discussion

### Transcriptome analysis of *A. commutatum* red valentine at the different leaf developmental stages

Even though several studies have been conducted on Red Valentine, they were mostly focused on phenotypic and biochemical characteristics [23–27], whereas none have investigated the molecular mechanisms behind the red coloration of Red Valentine foliage. The red colored leaves of Red Valentine are of considerable ornamental value and this trait is of interest to plant breeders. In the present study, we performed RNA-seq analysis of the leaves of *A. commutatum* Red Valentine and a green mutant to reveal their gene expression changes at three developmental stages and to identify the key genes involved in their leaf color variations. A total of 196.15 Gb clean data were obtained from 18 RNA-seq libraries. A total of 63,621 unigenes with an average length of 934 bp and an N50 length of 1340 bp were assembled. This number was similar to that in other *Araceae* species, such as *Amorphophallus konjac* [31], *Pinellia ternata* [32], and *Caladium hortulanum* [33]*.* The percentage of filtered Q30 and the GC content were higher than 94.71 and 50.54%, respectively. Xiong et al. [34] performed a transcriptome analysis of variegated citrus plants and reported a Q30 score higher than 94.19%. In another study on the transcriptome of calla lily spathes, the GC content was between 50.5 and 51.05% [35]. Thus, the quality of the RNA-seq data in the present study was good for further analyses.

### Variation in leaf anthocyanin and chlorophyll contents

Leaf color changes in ornamental plants are associated with the biosynthesis of pigments. Anthocyanin is a major pigment in plants and confers red, blue, and purple colors to the leaves, with chlorophyll being responsible for green leaf coloration [1, 36, 37]. In the Red Valentine variety of *A. commutatum*, we found that anthocyanins accumulated mainly in the palisade tissues, whereas chlorophylls were present in both the sponge and palisade tissues. At S3, the anthocyanin content of Red Valentine leaves was approximately 5.58-fold higher than that of the green mutant leaves. Contrarily, the chlorophyll content of the green mutant leaves was approximately 5.57-fold higher than that of Red Valentine leaves, which was in accordance with the leaf color phenotype of the two varieties. A previous HPLC analysis showed that cyanidin was the major anthocyanin component in the ever-red leaves of *Malus* cv. ‘Royalty’ [38]. Through metabolome analyses, cyanidin was proved to be the main contributor of the purple leaf phenotype in *Brassica napus* [39]. In the present study, cyanidin was found to be the major anthocyanin in the leaves of *A. commutatum*; however, the composition and proportion of anthocyanin monomers did not significantly affect the leaf color of *A. commutatum*. The results of our examination of the phenotypic and biochemical basis of changes in leaf color through the three developmental stages are vital for further molecular studies.

### Genes involved in anthocyanin and chlorophyll biosynthesis and degradation

RNA-seq technology is a powerful tool for revealing the relationship between different biological processes and gene expression, as well as for identifying key genes under various conditions [40]. Transcriptome analysis has been used to explore the mechanism underlying leaf color variation in many plant species, such as ornamental kale [41], *Paeonia qiui* [42], sweet basil [43], and *Acer truncatum* [44]. In the present study, we identified 283,130 unigenes in the leaves of *A. commutatum*. After filtering out low-expression unigenes, we identified 26 differentially expressed putative anthocyanin structural unigenes. Full-length cDNA of the genes was obtained, and phylogenetic trees were constructed to get more functional information for future studies. In accordance with the phenotype and anthocyanin content, the TPM values of most anthocyanin genes were significantly higher in the leaves of Red Valentine than in the leaves of the green mutant at the three developmental stages. Particularly, the TPM values of *CHS* (TRINITY_DN78614_c3_g2), *CHI* (TRINITY_ DN98854_c4_g1), *DFR* (TRINITY_DN75107_c2_g1, TRINITY_DN99635_c0_g4), and *UFGT* (TRINITY_ DN91923_c1_g1, TRINITY_DN90166_c0_g1) in Red Valentine leaves were more than 5-fold higher than those in the leaves of the green mutant at developmental stage S3.

Furthermore, we identified 31 unigenes involved in chlorophyll biosynthesis and 15 unigenes involved in chlorophyll degradation in the leaves of *A. commutatum*. The TPM values of these genes (Supplementary Table S4) were used to elucidate the gene expression pattern during the three developmental stages. Among the unigenes, the expression of 10 chlorophyll biosynthesis unigenes in the leaves of Red Valentine was 2-fold higher than that in the leaves of the mutant at the same developmental stage, indicating that these genes play vital roles in chlorophyll biosynthesis and accumulation. However, no significant differences in the expression profiles of the other chlorophyll biosynthesis and degradation unigenes were observed between the two varieties. Further research should be conducted to elucidate how chlorophyll biosynthesis and degradation genes influence these leaf color changes.

### TFs related to anthocyanin biosynthesis

Previous studies have revealed that anthocyanin biosynthesis is transcriptionally modulated by TFs, including R2R3-MYB, bHLH, and WD40. Among them, MYB TFs alone or in combination with MBW complexes (MYB, bHLH, and WD40) can control anthocyanin synthesis and accumulation [10, 45]. In the present study, two MYB TFs, namely AcMYB1 and AcMYB2, were identified as candidate anthocyanin biosynthesis regulators. These TFs are homologs of AaMYB1 and AaMYB2 in *A. andraeanum*, which were reported to be involved in anthocyanin regulation. Our phylogenetic analysis showed that the two TFs belonged to the same anthocyanin subgroup. Overexpression of AcMYB2 resulted in the red leaf phenotype of transgenic plants, indicating its anthocyanin regulatory activity in *A. commutatum* Red Valentine (Supplementary Fig. S5)*.* Transposon insertion and methylation of the promoter on R2R3-MYB TFs regulating anthocyanin biosynthesis have been reported in *Phalaenopsis* orchids [46], apple [47, 48], and pear [49]; they could directly control MYB TF expression, consequently affecting anthocyanin accumulation. The candidate unigenes exhibited significantly higher expression profiles in the Red Valentine variety than in the green mutant. Therefore, we speculated that transposon insertion or methylation of the AcMYB2 promoter may occur during leaf development, consequently decreasing or stopping the expression of *AcMYB2*, resulting in green mutants. Further studies should investigate the role of these key TFs in the regulation of leaf color.

Additionally, several other TFs have been reported to participate in anthocyanin biosynthesis. In *Arabidopsis* and *B. napus*, BnWRKY41–1 was found to have a similar role as that of AtWRKY41 in anthocyanin biosynthesis regulation [50]. In pear, ERFs can interact with PpMYB114 to regulate the light-dependent anthocyanin pathway in fruits [51]. NAC TFs were found to be potential regulators of anthocyanin biosynthesis in apple [52] and litchi [53] during fruit ripening. In the present study, a total of 1068 TFs belonging to 32 TF families were identified by aligning them to the PlantTFDB database using BLASTX. Among them, TRINITY_DN60809_c4_g1 (WRKYs), TRINITY_DN106460_c0_g1 (WRKYs), TRINITY_DN62456_c3_g4 (ERFs), and TRINITY_DN101050_c1_g1 (NACs) were screened as the candidate TFs regulating the anthocyanin pathway because their expression pattern was similar to that of anthocyanin-related genes.

### Effects of plant hormones on leaf color variation

Plant hormones play critical roles in stimulating anthocyanin accumulation. For example, abscisic acid (ABA) and jasmonates (JAs) can induce anthocyanin accumulation by regulating anthocyanin-related genes. In *Litchi chinensis*, LcABF1/2/3 were reported to be important ABA-dependent regulators in both chlorophyll degradation and anthocyanin biosynthesis [54]. After silencing a key gene in the ABA biosynthesis pathway of sweet cherry, the fruits showed a colorless phenotype [55]. Besides, the molecular mechanism underlying JA-regulated anthocyanin accumulation was first reported in *Arabidopsis* [56]. Jasmonate-ZIM-domain proteins disrupted the formation of WD-repeat/bHLH/MYB complexes to repress JA-regulated anthocyanin accumulation [57]. In the present study, a total of 354 unigenes were annotated to plant hormone signal transduction in the KEGG databases. Among them, more than 43 unigenes were identified as DEGs by pairwise comparison. The abundant gene expression and sequence data provided in the present study lays the foundation for exploring the molecular processes underlying the role of plant hormones in the leaf variation of *A. commutatum.*

## Conclusions

The present study demonstrated that high chlorophyll and low anthocyanin contents were responsible for the green leaf coloration of the *A. commutatum* green mutant. Furthermore, 26 anthocyanin biosynthesis structural genes and four key regulatory TFs, namely AcMYB1, AcMYB2, AsTTG1, and AcbHLH1, were identified. Downregulation of these TFs may downregulate the expression of anthocyanin biosynthesis genes. Further molecular studies are needed to elucidate the role of each TF and structural gene in anthocyanin biosynthesis in Red Valentine. The findings of the present study provide insights into anthocyanin candidate genes, which can facilitate the breeding and development of ornamental cultivars and contribute to the understanding of leaf color variation in *A. commutatum*.

## Methods

### Plant materials

In the present study, 1-year-old tissue culture seedlings of *A. commutatum* ‘Red Valentine’ and a green mutant were selected as experimental materials (Fig. 1). The plants were purchased from the Guangzhou Flowers Research Centre (Guangzhou, China; NCBI:txid1210880) and grown in a greenhouse covered with a 60% shade cloth at the South China Botanical Garden, Chinese Academy of Sciences (Guangzhou, China). The use of this plant material was licensed and approved by the Guangzhou Flowers Research Centre. The temperature and relative humidity of the greenhouse ranged from 15 to 34 °C and 75 to 99%, respectively. Different leaf developmental stages were defined as follows: stage 1 (S1), white curly leaves with small amount of pigment (7 d); stage 2 (S2), uncurled leaves exhibiting light pink coloration (28 d); stage 3 (S3): mature leaves with characteristic dark coloration and oily surface (35 d). All samples were stored at − 80 °C until further analysis in May 2019.

### Anthocyanin and chlorophyll extraction and observation

Total anthocyanin content of *A. commutatum* was determined using the pH difference method [58] with modifications. Briefly, methanolic extracts of fresh leaves were prepared. To improve the extraction efficiency, fresh leaves (1 g) were subjected to ultrasonic agitation for 0.5 h. Subsequently, the sample was incubated with 15 mL of methanol (containing 0.05% HCl) in darkness for 12 h at 0 °C. Absorbance at 510 nm and 700 nm was measured in triplicate using a microplate reader (Tecan Infinity, Switzerland). For quantifying anthocyanin monomers, total anthocyanins extracted from leaf samples were hydrolyzed using 3 mol/L muriatic acid for 1 h at 97 °C in darkness, after which the solution was dried using a vacuum thickener (Concentrator plus; Eppendorf) to obtain anthocyanin monomer powder. The powder was re-dissolved in acidic methanol (0.05% HCl) for HPLC analysis, with cyanidin chloride, peonidin chloride, delphinidin chloride, pelargonidin chloride, petunidin chloride, and malvidin chloride (Phyproof, Germany) as internal standards. The anthocyanin content was determined by linear regression and expressed in mg/g of fresh leaf weight. Total leaf chlorophyll content was determined following the procedures described in Lichtenthaler [59]. Briefly, approximately 1 g of leaf sample was extracted with 1 ml of methanol, and the absorbance was measured at 652 nm, 665 nm, and 470 nm using a microplate reader (Tecan Infinity).

Freehand sectioning of the leaves was performed for anatomical observation of the pigments and their localization in the leaf tissues of the two varieties at the three developmental stages using a digital microscope (DVM6; Leica, USA).

### RNA extraction, cDNA library construction, and sequencing

Total RNA was isolated from the leaves using an RNA kit (Polysaccharides & Polyphenolics-rich) (Hua Yueyang, Beijing, China) with RNase-free DNase I (TaKara) to eliminate genomic DNA contamination. Total RNA integrity and purity were determined using the 2100 Bioanalyzer (Agilent Technologies, Inc., Santa Clara, CA, USA) and quantified using the ND-2000 spectrophotometer (NanoDrop; Thermo Fisher Scientific, Wilmington, DE, USA). Only high-quality RNA samples were used for constructing the sequencing library. RNA purification, reverse transcription, library construction, and sequencing were performed by Shanghai Majorbio Bio-pharm Biotechnology Co., Ltd. (Shanghai, China) (Supplementary Fig. S1). Independent triplicate whole-leaf materials from Red Valentine (R) and a green mutant (G) at developmental stages S1, S2, and S3 were used to construct 18 cDNA libraries, which were named as R1_1, R1_2, R1_3, R2_1, R2_2, R2_3, R3_1, R3_2, R3_3, G1_1, G1_2, G1_3, G2_1, G2_2, G2_3, G3_1, G3_2, and G3_3. Libraries were prepared using an Illumina TruSeqTM RNA sample preparation kit (Illumina, San Diego, CA, USA) and sequenced on a flow cell using the Illumina HiSeq™ 6000 sequencing platform (Illumina).

### de novo assembly and annotation

High quality reads from the 18 libraries were assembled de novo using Trinity (http://trinityrnaseq.sourceforge.net/). To annotate the unigenes, the transcripts alignment was subjected against public databases, including the NCBI protein NR (http://www.ncbi.nlm.nih.gov), Clusters of Orthologous Groups (http://www.ncbi.nlm.nih.gov/COG), and KEGG databases (http://www.genome.jp/kegg), using BLASTx. To determine the processes the unigenes are involved in, GO function annotation was performed using the BLAST2GO software (http://www.blast2go.com/b2ghome). Metabolic pathway analysis was performed using the KEGG database [60].

### Identification of differentially expressed genes (DEGs) and functional enrichment analysis

To identify the DEGs between the two varieties, the expression level of each transcript was calculated according to the TPM method. RSEM (http://deweylab.biostat.wisc.edu/rsem/) [61] was used to quantify the gene abundance. Differential expression analysis was performed using DESeq2 [62], with a Q value ≤0.05. Unigenes with fold change > 2 or < − 2 and a Q value ≤0.05 were considered significantly differentially expressed. The DEGs were used for GO function enrichment analysis and KEGG pathway analysis using Goatools (https://github.com/tanghaibao/Goatools) and KOBAS (http://kobas.cbi.pku.edu.cn/home.do) [63].

### Quantitative RT-PCR analysis

Total RNA (the same sample used for RNA-seq) was reverse-transcribed to first-strand cDNA for qRT-PCR using a TransScript® One-Step gDNA Removal cDNA Synthesis SuperMix (Transgen, Beijing, China). Diluted cDNA was amplified with specific primers (listed in Additional file) and reacted with PerfectStart Green qPCR SuperMix (Transgen) on a LightCycler 480 II (Roche) under the following conditions: 45 cycles at 94 °C for 5 s, 57 °C for 15 s, and 72 °C for 10 s. The gene expression levels were normalized using *A. commutatum* Red Valentine actin gene as the internal control and calculated using the 2^-ΔΔCT^ method. Three biological replicates were used for the analysis.

## Supplementary Information

**Additional file 1: Supplementary Fig. S1.** Functional categories of all unigenes in *A*. *commutatum* “Red Valentine” RNA-seq database. A. Gene Ontology (GO) classification of unigenes. All annotated unigenes were divided into three functional GO categories: biological process (BP), cellular component (CC) and molecular function (MF); B. Kyoto Encyclopedia of Genes and Genomes (KEGG) pathway classification. **Supplementary Fig. S2.** Melting curves of the reference genes actin (ACT) and 12 qRT-PCR genes involved in anthocyanin biosynthesis and regulation. *PAL: phenylalanine ammonia-lyase; C4H: trans-cinnamate 4-monooxygenase; 4CL: 4-coumarate--CoA ligase 2; CHS: chalcone synthase; UFGT: UDP-flavonoid glucosyl transferase; F3’H: flavanone 3-hydroxylase; DFR: dihydroflavonol 4-reductase.***Supplementary Fig. S3.** Phylogenetic trees of transcription factors and structural gene related to anthocyanin biosynthesis in *A*. *commutatum* “Red Valentine”. The phylogenetic trees were constructed using Neighbor-jonining of MEGA software (Molecular Evolutionary Genetics Analysis version 7.0, Kumar et al., 2016) with 1000 bootstrap replicates. *CHI: chalcone isomerase; F3H: flavanone 3-hydroxylase; ANS: anthocyanidin synthase.***Supplementary Fig** . **S4.** The phenotype of AsMYB2 transgenic tobacco with 35s promotor. A. Red leaf phenotype of transgenic plants overexpressing AsMYB2 against control plants. B. T-DNA region of the vector used in transgenic plants. LB: LB T-DNA repeat; NOST: NOS terminator; BlpR: phosphinothricin acetyltransferase, NOSP:NOS promoter, 35S: CaMV 35S promoter; mGFP: green fluorescent protein; EcoR I, Xba I: restriction site; RB: RB T-DNA repeat. **Supplementary Table S1.** Primers used in reverse transcription quantitative polymerase chain reaction (RT-qPCR) analysis. **Supplementary Table S2.** Data used in transcription factor identification. 26 putative anthocyanin biosynthesis unigenes were identified in RNA-seq database, and then normalized the exact PKM value in two varieties at there stages to reduce the error caused by the exact means using the free online platform of Majorbio Cloud Platform (www.majorbio.com), the normalized data were list in the table. **Supplementary Table S3.** The PKM value of unigenes connected with chlorophyll biosynthesis and degradation. **Supplementary Table S4.** Protein sequence of transcription factors and structural gene related to anthocyanin biosynthesis.

## Data Availability

All data generated or analyzed during this study are included in this published article and the supplementary information files. The sequence data was deposited in the NCBI database under the BioProject PRJNA793608.

## Abbreviations

CDS: Coding sequence
FC: Fold change
qRT-PCR: Quantitative real-time polymerase chain reaction
RACE: Rapid-amplification of cDNA ends
TPM: Transcript per million reads
