# Supplementary material for: Transcriptomic analysis reveals biosynthesis genes and transcription factors related to leaf anthocyanin biosynthesis in Aglaonema commutatum
Source: BMC Genomics. 2023 Jan 17;24:28. doi: 10.1186/s12864-022-09107-1 (PMC9847206; doi:10.1186/s12864-022-09107-1)
Supplement: Supplementary file 1 — Additional file 1: Supplementary Fig. S1. Functional categories of all unigenes in A. commutatum “Red Valentine” RNA-seq database. A. Gene Ontology (GO) classification of unigenes. All annotated unigenes were divided into three functional GO categories: biological process (BP), cellular component (CC) and molecular function (MF); B. Kyoto Encyclopedia of Genes and Genomes (KEGG) pathway classification. Supplementary Fig. S2. Melting curves of the reference genes actin (ACT) and 12 qRT-PCR genes involved in anthocyanin biosynthesis and regulation. PAL: phenylalanine ammonia-lyase; C4H: trans-cinnamate 4-monooxygenase; 4CL: 4-coumarate--CoA ligase 2; CHS: chalcone synthase; UFGT: UDP-flavonoid glucosyl transferase; F3’H: flavanone 3-hydroxylase; DFR: dihydroflavonol 4-reductase.Supplementary Fig. S3. Phylogenetic trees of transcription factors and structural gene related to anthocyanin biosynthesis in A. commutatum “Red Valentine”. The phylogenetic trees were constructed using Neighbor-jonining of MEGA software (Molecular Evolutionary Genetics Analysis version 7.0, Kumar et al., 2016) with 1000 bootstrap replicates. CHI: chalcone isomerase; F3H: flavanone 3-hydroxylase; ANS: anthocyanidin synthase.Supplementary Fig . S4. The phenotype of AsMYB2 transgenic tobacco with 35s promotor. A. Red leaf phenotype of transgenic plants overexpressing AsMYB2 against control plants. B. T-DNA region of the vector used in transgenic plants. LB: LB T-DNA repeat; NOST: NOS terminator; BlpR: phosphinothricin acetyltransferase, NOSP:NOS promoter, 35S: CaMV 35S promoter; mGFP: green fluorescent protein; EcoR I, Xba I: restriction site; RB: RB T-DNA repeat. Supplementary Table S1. Primers used in reverse transcription quantitative polymerase chain reaction (RT-qPCR) analysis. Supplementary Table S2. Data used in transcription factor identification. 26 putative anthocyanin biosynthesis unigenes were identified in RNA-seq database, and then normalized the exact PKM value in two varieties at [file 12864_2022_9107_MOESM1_ESM.pdf]

Supplementary Fig. S1. Functional categories of all unigenes in *A. commutatum* "Red Valentine" RNA-seq database. A. Gene Ontology (GO) classification of unigenes. All annotated unigenes were divided into three functional GO categories: biological process (BP), cellular component (CC) and molecular function (MF); B. Kyoto Encyclopedia of Genes and Genomes (KEGG) pathway classification.

A

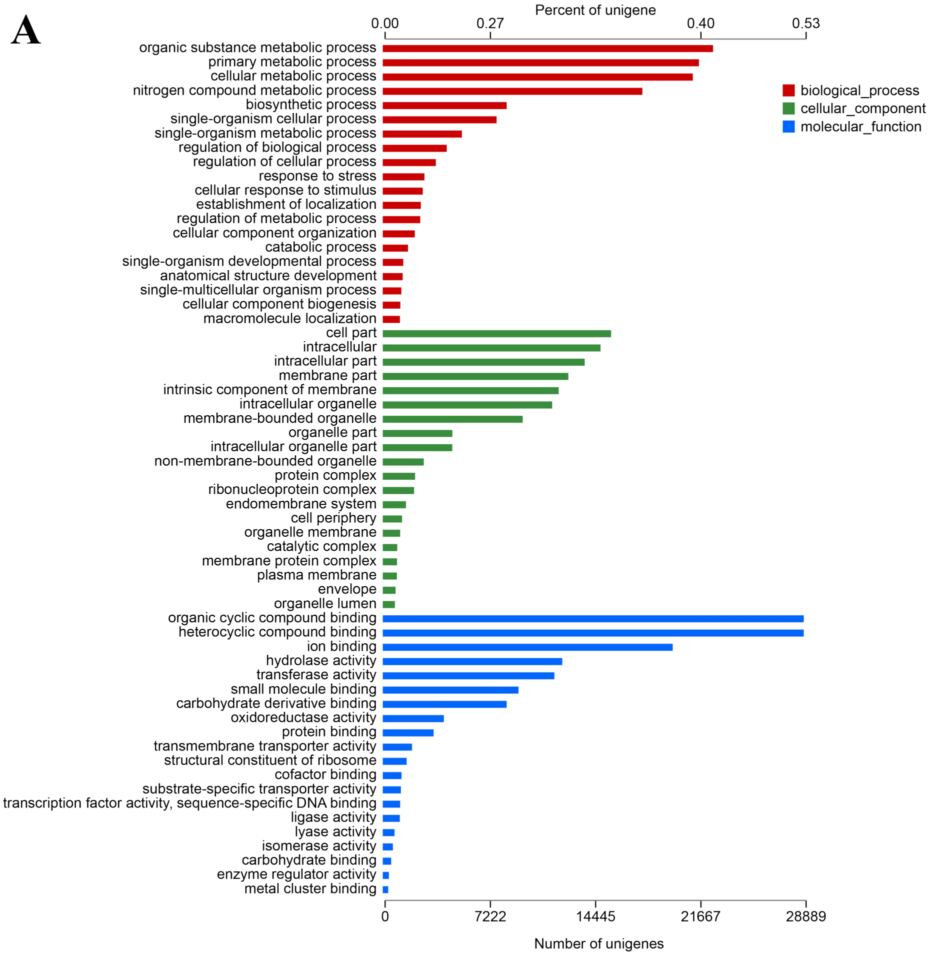

B

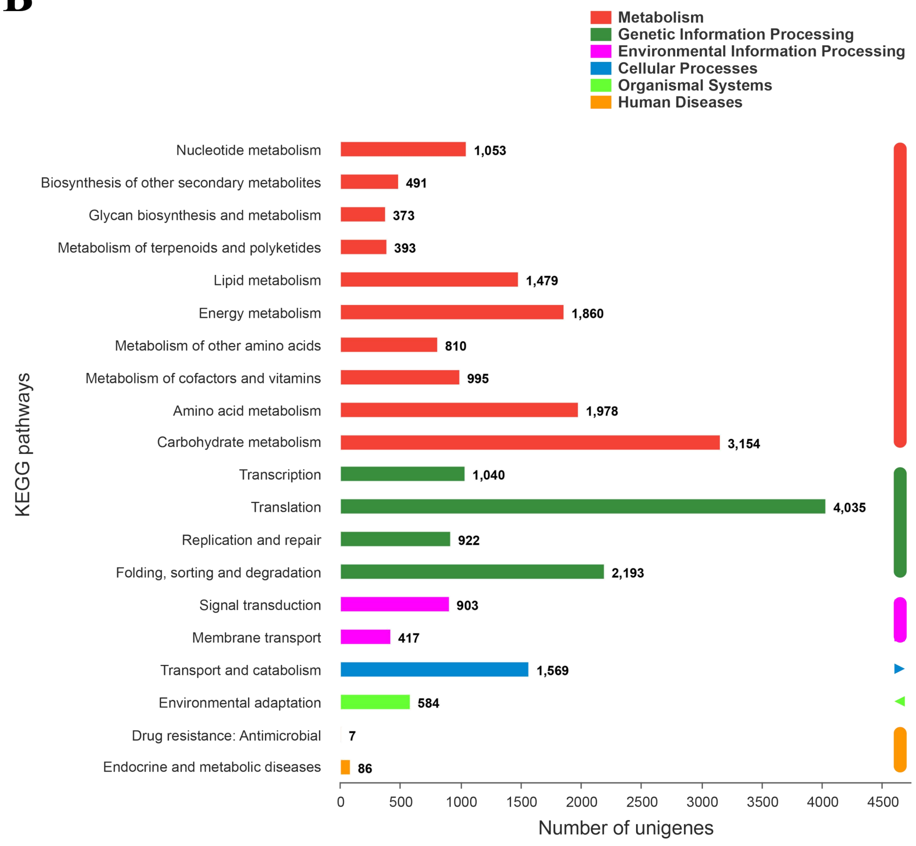
Metabolism
Genetic Information Processing
Environmental Information Processing
Cellular Processes
Organismal Systems
Human Diseases
KEGG pathways
Nucleotide metabolism
1,053
Biosynthesis of other secondary metabolites
491
Glycan biosynthesis and metabolism
373
Metabolism of terpenoids and polyketides
393
Lipid metabolism
1,479
Energy metabolism
1,860
Metabolism of other amino acids
810
Metabolism of cofactors and vitamins
995
Amino acid metabolism
1,978
Carbohydrate metabolism
3,154
Transcription
1,040
Translation
4,035
Replication and repair
922
Folding, sorting and degradation
2,193
Signal transduction
903
Membrane transport
417
Transport and catabolism
1,569
Environmental adaptation
584
Drug resistance: Antimicrobial
7
Endocrine and metabolic diseases
86
0
500
1000
1500
2000
2500
3000
3500
4000
4500
Number of unigenes

Supplementary Fig. S2. Melting curves of the reference genes actin (ACT) and 12 qRT-PCR genes involved in anthocyanin biosynthesis and regulation. *PAL: phenylalanine ammonia-lyase; C4H: trans-cinnamate 4-monooxygenase; 4CL: 4-coumarate--CoA ligase 2; CHS: chalcone synthase; UFGT: UDP-flavonoid glucosyl transferase; F3'H: flavanone 3-hydroxylase; DFR: dihydroflavonol 4-reductase.*

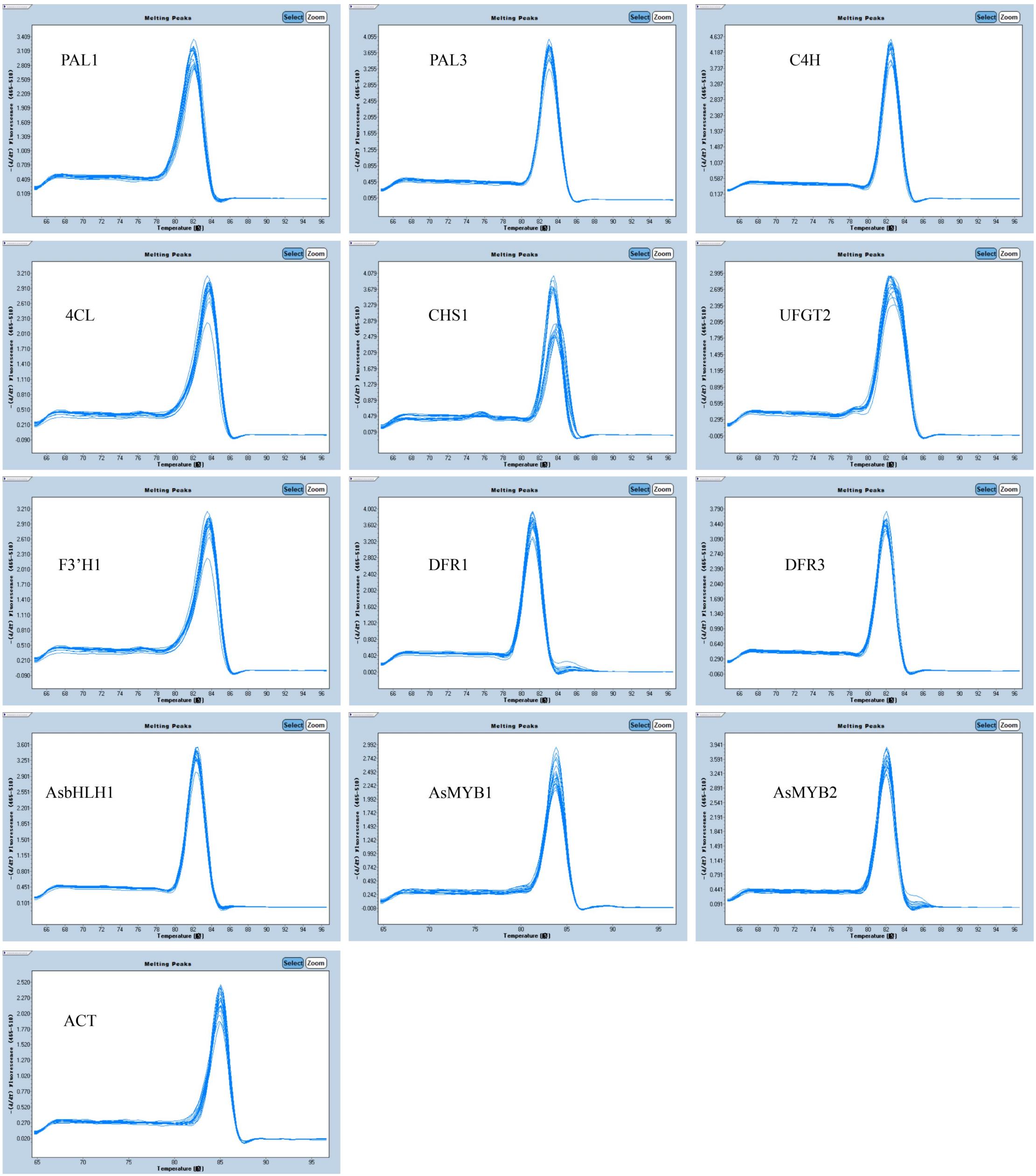

Supplementary Fig. S3. Phylogenetic trees of transcription factors and structural gene related to anthocyanin biosynthesis in *A*. *commutatum* “Red Valentine”. The phylogenetic trees were constructed using Neighbor-jonining of MEGA software (Molecular Evolutionary Genetics Analysis version 7.0, Kumar et al., 2016) with 1000 bootstrap replicates. *CHI: chalcone isomerase; F3H: flavanone 3-hydroxylase; ANS: anthocyanidin synthase.*

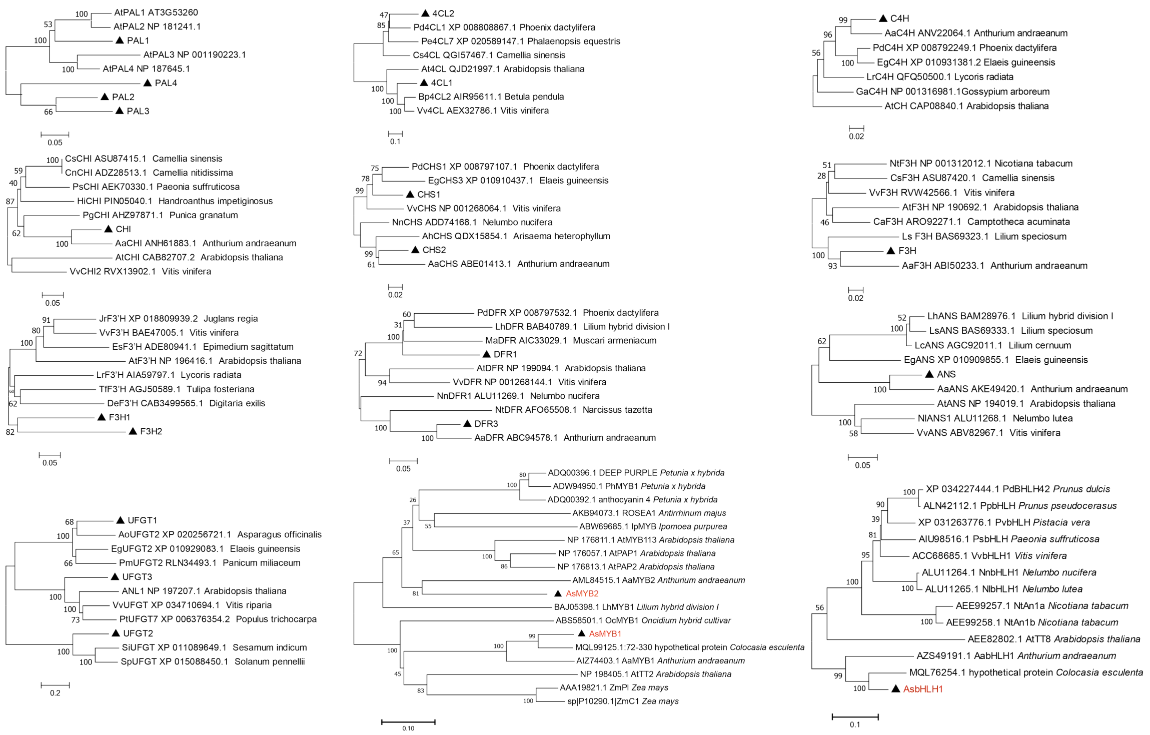

Supplementary Fig . S4. The phenotype of AsMYB2 transgenic tobacco with 35s promotor. A. Red leaf phenotype of transgenic plants overexpressing AsMYB2 against control plants. B. T-DNA region of the vector used in transgenic plants. LB: LB T-DNA repeat; NOST: NOS terminator; BlpR: phosphinothricin acetyltransferase, NOSP:NOS promoter, 35S: CaMV 35S promoter; mGFP: green fluorescent protein; EcoR I, Xba I: restriction site; RB: RB T-DNA repeat.

A

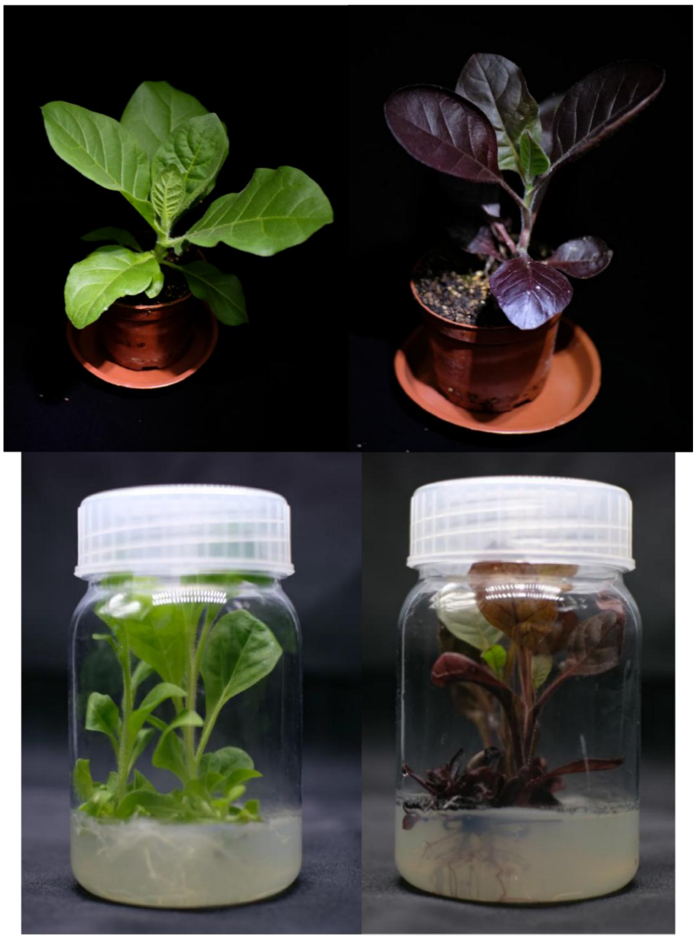

Control AsMYB2-L1

B

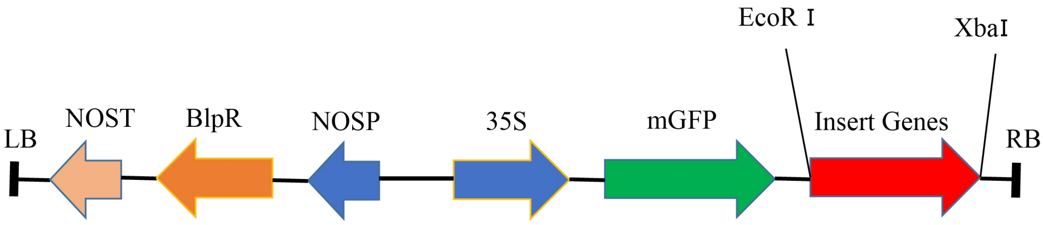

Supplementary Table S1. Primers used in reverse transcription quantitative polymerase chain reaction (RT-qPCR) analysis.

Gene	Unigene ID	Forward primer (5'-3')	Reverse primer (5'-3')
ACT	TRINITY_DN92045_c1_g1	GCCGCCCCCATATTAAGTGA	GACGGCTCTATCCAAGCCAG
PAL1	TRINITY_DN98590_c0_g3	TCTCGCGTTCGATGGACTTG	AAGCCTAAGCAGGACCGCTA
PAL3	TRINITY_DN89524_c3_g6	GATGCTCGCGTTGAGGAAGT	CTACGGCGTGACTACCAACT
C4H	TRINITY_DN95047_c0_g1	CCTGCAGATAGGGGAGCTTG	GAGACGACGTTGTGGTCGAT
4CL	TRINITY_DN95116_c0_g1	CTACGGGATGACGGAGGC	ATGATCTGAGCTCCCCGGAT
CHS1	TRINITY_DN78614_c3_g1	CCGTACTCCTTGAGCACCTC	GGGCATCAACGACTGGAACT
UFGT2	TRINITY_DN90166_c0_g1	CTTCTTCTCCGTCTTCGCCG	AAGTCAGCGGCCTGGTAAAG
F3'H1	TRINIY_DN103984_c0_g1	GAGAGGCACCGACTTTGAGG	CTGCAAGGTGAGTCCGTAGG
DFR1	TRINITY_DN75107_c2_g1	GCTGAGCGTATTGAGCTCCT	CATCCAGCCGGTCATCTTGA
DFR3	TRINITY_DN99635_c0_g4	AGATGTACCTTCCGCTGGCT	TCTAATTTGGATGGCCCCCTG
AsbHLH	TRINITY_DN107490_c2_g1	TGGCTTTCAGGGGCAAATGA	GTCCTCGTCCACCTTATCGG
AsMYB1	TRINITY_DN91028_c1_g1	GGCTCAACTACCTCAGACCG	AAGGACCACCTGTTGCCAAT
AsMYB2	TRINITY_DN62889_c2_g2	TGCTCCTACTGTGCAACCTT	TTGCTCGTCGTTTCCCTCAG

Supplementary Table S2. Data used in transcription factor identification. 26 putative anthocyanin biosynthesis unigenes were identified in RNA-seq database, and then normalized the exact PKM value in two varieties at there stages to reduce the error caused by the exact means using the free online platform of Majorbio Cloud Platform (www.majorbio.com), the normalized data were list in the table.

Gene ID	R1	R2	R3	G1	G2	G3
TRINITY_DN103984_c0_g1	0.0635	1.4302	0.5109	0.2005	-0.3222	-1.8829
TRINITY_DN105397_c3_g1	-1.4951	0.5422	1.5370	-0.9361	-0.1157	0.4676
TRINITY_DN64962_c0_g1	-0.0395	1.1772	0.9478	-0.2078	0.0380	-1.9156
TRINITY_DN72679_c3_g2	-0.6362	1.2224	1.2901	-1.5314	-0.0465	-0.2985
TRINITY_DN75107_c2_g1	-0.1222	1.4176	0.9334	-1.3294	0.2331	-1.1325
TRINITY_DN78614_c3_g1	-0.2124	1.5022	0.6651	0.2392	-0.4679	-1.7263
TRINITY_DN78614_c3_g2	-0.6858	1.4068	1.2651	-1.1254	-0.0347	-0.8261
TRINITY_DN79161_c1_g2	-0.4868	1.6355	0.9138	-0.3524	-0.2776	-1.4324
TRINITY_DN81970_c0_g2	-0.2083	1.1365	1.0791	0.0330	-0.1782	-1.8622
TRINITY_DN84294_c0_g1	0.2258	1.4983	0.6467	-0.2457	-0.3671	-1.7580
TRINITY_DN89524_c3_g6	-0.2062	1.5062	0.9751	-1.1611	0.0633	-1.1773
TRINITY_DN90166_c0_g1	0.0432	1.4706	0.8455	-1.1114	0.1199	-1.3679
TRINITY_DN91923_c1_g1	-0.0708	1.4234	1.1266	-1.0461	-0.1786	-1.2544
TRINITY_DN93899_c0_g3	-0.1495	1.5601	0.8295	-0.3694	-0.2393	-1.6315
TRINITY_DN95047_c0_g3	-1.1106	0.0795	2.0114	-0.1339	-0.0119	-0.8344
TRINITY_DN95116_c0_g1	-0.2223	1.2928	1.0456	-0.3479	-0.0175	-1.7506
TRINITY_DN98590_c0_g2	-0.9606	1.7063	0.8840	-1.0277	-0.0296	-0.5723
TRINITY_DN98590_c0_g3	-0.3607	1.5693	0.8708	-0.3602	-0.1381	-1.5811
TRINITY_DN98590_c0_g4	-0.3412	1.5440	0.8765	-0.2267	-0.2322	-1.6205
TRINITY_DN99635_c0_g4	-0.2998	1.3339	1.1741	-1.1794	0.1305	-1.1593
TRINITY_DN106976_c3_g1	0.7398	1.4708	-0.7318	0.3369	-0.2036	-1.6121
TRINITY_DN74649_c0_g2	-0.1611	1.8531	-0.7847	-0.8083	0.7461	-0.8452
TRINITY_DN85368_c0_g3	-0.2086	1.6245	-1.6566	-0.0336	0.6541	-0.3798
TRINITY_DN93219_c1_g2	0.7917	0.0891	-0.5925	1.5896	-0.3374	-1.5406
TRINITY_DN95047_c0_g1	0.3902	1.2819	0.3401	0.3773	-0.4560	-1.9335
TRINITY_DN98854_c4_g1	0.5280	0.8612	0.1962	0.7199	-0.2130	-2.0922

Average	-0.1998	1.2937	0.6615	-0.3861	-0.0724	-1.2969

Supplementary Table S3. The PKM value of unigenes connected with chlorophyll biosynthesis and degradation.

HEMA: glutamyl-tRNA reductase;

HEMB: aaminolevulinic acid dehydratase;

HEMC: porphobilinogen deaminase;

HEMD: uroporphyrinogen-III synthase;

HEME: uroporphyrinogen decarboxylase;

HEMF: coproporphyrinogen III oxidase;

HEMG: oxygen-dependentprotoporphyrinogen oxidase;

CHLH/D/I: magnesium chelatase subunit H/D/I

CHLM: magnesium protoporphyrin DC methyltransferase

CRD(ACSF): magnesium-protoporphyrin IX monomethyl ester (oxidative) cyclase ;

PORA/B/C: protochlorophyllide reductase A/B/C;

DVR: divinyl chlorophyllide a 8-vinyl-reductase;

CAO: chlorophyllide a oxygenase;

NYC1/NOL: chlorophyll(ide)b reductase;

HCAR: 7- hydroxymethyl chlorophyll a reductase;

SGR: stay-green;

PPH: pheophytin pheophorbide hydrolase

PAO: pheophorbide a oxygenase;

RCCR: red chlorophyll catabolite reductase;

	Gene	Unigene ID	G1	G2	G3	R1	R2	R3
Chlorophyll biosynthesis and cycle related genes	*HEMA*	TRINITY_DN63347_c1_g1	48.71	46.52	43.04	53.34	54.68	49.43
		TRINITY_DN95832_c0_g1	15.48	17.51	12.72	18.32	27.08	14.02
		TRINITY_DN63567_c0_g1	46.09	81.58	76.38	45.62	42.92	32.80
		TRINITY_DN95832_c0_g2	67.72	96.35	97.28	47.32	50.98	42.84
		TRINITY_DN74857_c0_g3	67.37	58.40	51.41	52.42	45.81	52.56
		TRINITY_DN83538_c2_g3	45.01	36.59	33.63	36.92	33.52	36.79

	HEMB	TRINITY_DN98110_c0_g2	145.93	161.86	201.20	119.51	83.39	104.82
	HEMC	TRINITY_DN67480_c5_g1	323.86	253.02	150.97	268.48	145.90	117.76
	HEMD	TRINITY_DN78090_c2_g1	3.12	4.98	8.82	3.43	3.86	5.00
		TRINITY_DN93739_c1_g1	94.34	107.58	79.97	98.45	105.43	70.24
	HEME	TRINITY_DN101863_c2_g3	7.56	11.46	17.04	7.53	8.20	10.88
		TRINITY_DN103757_c0_g1	45.16	59.43	33.03	48.02	18.85	45.71
		TRINITY_DN66202_c2_g2	61.06	64.17	48.76	52.26	49.43	46.53
		TRINITY_DN76425_c1_g1	5.77	8.07	4.34	4.72	3.83	5.78
		TRINITY_DN101863_c2_g2	3.72	7.30	5.85	3.47	1.02	4.08
	HEMF	TRINITY_DN63968_c0_g1	6.88	3.73	5.29	3.12	3.26	3.13
		TRINITY_DN63968_c0_g4	191.91	167.99	120.25	132.88	97.91	107.24
		TRINITY_DN96909_c1_g1	172.35	166.96	115.40	139.25	102.38	90.52
	HEMG	TRINITY_DN105042_c2_g1	9.10	6.40	4.75	7.00	5.18	6.29
	CHLH/D/I	TRINITY_DN95468_c3_g1	4.64	10.80	11.62	2.84	3.83	3.69
		TRINITY_DN95468_c3_g6	6.33	5.85	7.61	5.35	5.64	4.53
		TRINITY_DN96966_c1_g3	12.45	28.24	35.59	6.76	9.46	9.04
	CHLM	TRINITY_DN78841_c1_g1	0.77	0.88	0.60	0.73	0.73	0.98
	CRD(ACSF)	TRINITY_DN88221_c0_g1	7.91	13.74	7.91	5.29	6.54	2.13
		TRINITY_DN78858_c3_g1	413.35	838.30	729.50	317.91	288.23	293.31
	PORA/B/C	TRINITY_DN93340_c1_g1	476.90	492.65	554.98	264.70	158.36	231.61
	DVR	TRINITY_DN90068_c2_g2	41.29	63.26	51.88	34.89	40.26	30.10
		TRINITY_DN101935_c0_g2	191.89	204.07	132.38	212.14	149.98	131.70
	CAO	TRINITY_DN58016_c0_g1	0.76	0.68	0.80	1.39	1.52	3.03
		TRINITY_DN86926_c2_g1	13.21	17.88	16.02	6.46	3.73	4.99
		TRINITY_DN87057_c1_g1	25.21	18.14	11.72	13.30	4.89	5.72
	NYC1/NOL	TRINITY_DN83767_c1_g1	29.04	26.03	30.12	24.91	14.61	18.93
		TRINITY_DN101603_c0_g1	59.47	104.45	121.06	72.11	93.30	95.77
Chlorophyll degradation related genes	*HCAR*	TRINITY_DN103561_c3_g1	3.98	3.74	4.86	2.38	3.51	5.51
		TRINITY_DN89117_c0_g1	26.76	21.10	29.53	16.99	26.92	30.74
		TRINITY_DN89117_c0_g2	41.80	51.99	52.00	32.89	39.21	46.98
	SGR	TRINITY_DN83555_c0_g1	2.66	0.96	3.07	3.82	7.23	5.90
	PPH	TRINITY_DN81824_c0_g5	20.30	42.86	70.84	21.64	42.42	59.38
		TRINITY_DN89834_c0_g4	27.15	34.90	78.43	27.00	36.04	54.02

	TRINITY_DN96660_c0_g1	10.89	23.83	21.31	10.08	15.55	10.91
	TRINITY_DN79574_c1_g1	42.60	52.89	75.21	37.32	48.95	46.62
	TRINITY_DN96584_c1_g1	39.81	65.30	117.65	44.95	72.35	85.03
PAO	TRINITY_DN96961_c1_g1	36.50	58.61	77.46	32.06	48.45	64.67
	TRINITY_DN88053_c0_g6	44.54	112.65	219.90	45.54	96.62	151.59
	TRINITY_DN88961_c5_g8	6.75	13.17	17.33	9.40	13.69	15.65
RCCR	TRINITY_DN85771_c0_g1	69.81	119.19	136.00	85.47	107.34	115.52

Supplementary Table S4. Protein sequence of transcription factors and structural gene related to anthocyanin biosynthesis.

>PAL1
MEAAPQANCNGHANGTVESFCLKPAVVAYGDPLNWGVAAESLKGSHLDAVKRMVDEFRRPLVRLEGADLKISQVAA
VAASGSGIKVELAESARAGVKASSEWVMDSMNKGTDSYGVTTGFGATSHRRTKQRGALQKELIRFLNAGIFGSGPDYG
NTTLPPTATRAAMLVRVNTLLQGYSGIRFEILEAITAFLNHNVTPCLPLRGTITASGDLVPLSYIAGMLTGRPNAKALTTD
GHHVSADEAFRLAGIPGFFDLQPKEGLALVNGTAVGSGLASMVLFEANILAVLAEVLSAVFCEVMQGKPEYTDHLTHK
LKHHPGQIEAAAIMEHILDGSSYVKMAKRLHELDPQQKPKQDRYALRTSPQWLGPQVEVLRQATKSIEREINSVNDNPL
IDVSRSKALHGGNFQGTPIGVSMDNARLAIAAIGKLMFAQISELVNDFYNNGLPSNLSGGRNPSLDYGFKGAEIAMAAY
CSELQFLGNPVTNHVQSAEQHNQDVNSLGLISARKTAEAVEILKLMASTYMVALCQAVDLRHLEENLKNAVKNTVSQ
VAKRVLTMGPNGELHPSRFCEKDLIRAVDREHVFTYADDPCSASYPLMQKLRQVLVEHALGNGEKEKDANTSIFQNIA
AFEEELQMALPKEVEAARVAFENGTSATPNRIEECRSYPLYRFVRAELGTSMLTGEKVRSPGEDFDKVFVAISQGKVVD
PLFECLKGWNGAPIPIC

>PAL2
MATKSNGLTCPTDAFRPPTLPLPKNGINPIFVSGSSYIPNPPHWKKAAEALESTHFEEVRRMISQYQATQAVDLQGTTLT
VAQVAAVARLSNVAVRLNEAVAKERVAKSANWVADNIARGTDTYGVTTGFGATSHRRTKKVTDLQTELIRFLNAGVI
GKEHLPSSYAKAAMLVRTNTLMQGYSGIRWEILEAITKLMNENIIPKLPLRGTITASGDLVPLSYIAGLLTGRHNSRAVT
PEGEEITSAEALKRVGIEAPFALQAKEGLALVNGTAVGSAVAATVCYDANILALLSVVLSALFCEAMQGKPEFADPLTH
ELKHHPGQIESAAIMEFLLDGSDYMREAKIRNEREPLTKPKQDRYALRTSPQWLGPQIEVIRMATHAIEREINSVNDNPLI
DVAGDRAVHGGNFQGTPVGVSMDNTRLALAAIGKLMFAQFTELVNDFYSNGLPSNLSAGPDPSLDYGFKGAEIAMAA
YTSELQFLANPVTNHVQSAEQHNQDVNSLGLISARKSEEAIHILQLMSATYIVALCQAVDLRHAEENLKHTVKHVISQV
AKRVLSVGSNGELLESRFWEKKLLKLVESEHVFNYIDDPCSSDYPLMQKLRQVLVEQAMASPDKERSERASILRRMLEF
EEEVKALLPRGVEATREALEKGKAMIPNRIEQCRSYPMYQFVRTELNTVVISGARILPPGEDFDKVFVAISEGKLVDPLL
SCLEEWDGSPLPVW

>PAL3
MEDHPVSNGRKHSQEGDPSALSPPHSTDPLCWGASADALRGSHLEQVKKLVSQFRKPHIELAGSGLTVAHVAAVARSS
TCTVALCERKRVLVEASSKWVCESACNGVDSYGVTTGFGANSHRRTNQSEALQTELVRFLNAGILAGATPLPAPATRA
AMLVRANTLLQGYSGIRWEILQAIAAMLNHGVTPLVPLRGSITASGDLLPLSYVAGVLTGRPNARAVLPDGAVVGAEE
ALRRAGVATGCFKLHAKEGLALVNGTAAGAGLAAMVLYDANELALLASVVSALFCEVMLGKPQFTDHLTHRLKHHP
GQIEAAAIMEHVLQGSSYMKRAQKHHETDPHLKPKQDCYALRTSPQWLGPQLEVIRAATASVGREINSVNDNPLIDVA
GDRAVHGGNFQGTPVGVSMDNTRLALAAIGKLMFAQFTELVNDFYSNGLPSNLSAGPDPSLDYGFKGAEIAMAAYTSE
LQFLANPVTNHVQSAEQHNQDVNSLGLISARKSEEAIHILQLMSATYIVALCQAVDLRHAEENLKHTVKHVISQVAKRV
LSVGSNGELLESRFWEKKLLKLVESEHVFNYIDDPCSSDYPLMQKLRQVLVEQAMASPDKERSERASILRRMLEFEEEV
KAVLPREVEATREALEKGKAMIPNRIEQCRSYPMYQFVRTELKTGVISGARILSPGEDFDKVFMAISEGKLVDPLLSCLE
EWDGSPLPVW

>PAL4
MAAKSNALTCPTDAFRPPTLPLPKNGINPIFVSGSSYIPNPPHWKKAAEALESTHFEEVRRMISQYQATQAVDLQGTTLT
VAQVAAVARLSNVAVRLNEAVAKERVAKSANWVADNIARGTDTYGVTTGFGATSHRRTKKVTDLQTELIRFLNAGVI
GKEHLPSSYAKAAMLVRTNTLMQGYSGIRWEILEAITKLMNENIIPKLPLRGTITASGDLVPLSYIAGLLTGRHNSRAVT
PEGEEITSAEALKRVGIEAPFALQAKEGLALVNGTAVGSAVAATVCYDANILALLSVVLSALFCEAMQGKPEFADPLTH
ELKHHPGQIESAAIMEFLLDGSDYMREAKIRNEREPLTKPKQDRYALRTSPQWLGPQIDVIRMATHAIEREINSVNDNPLI
DVARDIALHGGNFQGTPVGVSMDNLRIALAAIGKLVFAQFSELICDYYNNGLPSNLSGGADPSLDYGLKGAEIAMAAY
CSELQYLANPVTTHVQSAEQHNQDVNSLGLISARKSAEAVEILKLMVSTYMVGLCQALDLRHREENLREVVKHIVAQA
ARKTLYTAEDGLMLESRFCEKELLQVVECQPVFSYIDDPANPSYALLLQLREVLVEKSLNKSEDGYAVFKRIPVFQEEL
RRTLSEEVPRARERFEKGDFLVGHRIKQCRTYPVYKLVRSEAGAAFLTGEKQVSPGECIEKVVDAIGEGKLGEALNGCL
AMWRGSAGPFTPRPEVSSPAHCNPEFWSWFDKVRSPSATSGRGYWNQ

>C4H
MDLVFLEKVLLGLFVAVMLAIAVSKARGKRFKLPPGPLPVPVFGNWLQVGDDLNHRNLTALARRFGDIFLLRMGQRN
LVVVSSPDLARDVLHTQGVEFGSRTRNVVFDIFTGKGQDMVFTVYGDHWRKMRRIMTVPFFTNKVVQQYRFGWEDE
ARRVVEDIRADPKAATEGVVLRRRLQLMMYNNMYRIMFDRRFESEEDPLFVKLRALNGERSRLAQSFEYNYGDFIPILR
PFLRGYLKICKEVKERRLQLFKDYFLEERKRIGSTKGSDGLKCAIDHILDAEKKGEINEDNVLYIVENINVAAIETTLWSI
EWGIAELVNHPEIQRKLRSEIEKVLGPGAQVTEPDTYKLPYLQAVIKETLRLRMAIPLLVPHMNLHDAKLGGYCIPAESK
ILVNAWYLANNPDMWKRPEEFRPERFLEEEAKVEANGNDFRYLPFGVGRRSCPGIILALPILGITIGRLVQNFELLPPPGH
DKLDTSEKGGQFSLHILKHSTIVAKPRVF

>4CL1
MLSVAARENQNQVASEGLPPRPPAPETTIFRSKLPDIPIPDHLVLHDYCFERAAEFADRPCLIAGATGRTYTFAETHLLCR
RTAAGLAVLGVGRGDTIMLLVQNCPEFAFAFMAASMLGAKATTANPFCTPAEIAKQFRSSGAKLVVTQAQFVGTLRCE
LFPKVGEGMAVVTVDEQPAPEGCVSFSDVSGADEGAVPEVGIGPDDAVALPFSSGTTGLPKGVMLTHRSLVTNVAQQV
DGDNPNLHLGAGDVVLCVLPLFHIFSLNSVLLCSLRAGAAVLVVPKFEIAGLLGLIQRHRVTVAAVVPPIVLALAKNPT
VESFDLSSIRIVLSGAAPLGSELEEALKNRVPQATFGQGYGMTEAGPVLTMCPAFAKEPLPAKSGSCGNVVRNAELKVI
DPETGFSLRRNQPGEICIRGAQIMKGYLDDPAATAATIDVDGWLHTGDIGYVDDDDEVFIVDRVKELIKFKGFQVPPAE
LEALLISHPSIADAAVVPAMDEAAGEVPVAFVVRSNGFELTEDAVKEFVAKQVVYYKRLHKVYFTPAIPKSPSGKILRK
DLRAKLATHA

>4CL2
MANSSGYCSRSGIYRSPRPPLPFPPDSDLSMVRFLLRNAASHPDRLALADADADSGRCLTFRELSPAIHRVAAAISRLGV
GKGDVVLLFAPNSLDFPLCFLGAIHLGAVVTTVNPAYTVPELSKQAADSGAKVVITVPQLWPKVMALRLPTVITGPDRV
AVAPGDGAGTEIPAVAYLPDLLDSVGDAPDFVPPPVRQGDVAALFYSSGTTGANKGVILTHRNFIVASLMVTADDDRR
GEPPHVSLCFLPMFHIYGMALVAYAQLRRGGAVVSMVRFEMEAVLRAVERYRVSSVSLVPPVMIALAKQGKVTRYNL
SSLRQITTGAAPVGKDTLEEVSRLYPGVQVIQGYGLTETCGIISIEIPGEGGRKFGSTGNLVPGVEAKVTSVDTLKPLPPN
QLGELCFRGPNIMQGYFNNPQATKLTLDEEGWLHTGDLGYFDDQGLLFVVDRIKELIKYKGFQVAPAELEGLLLSHPEI
LDAAVIPFPDEEAGEVPVAYVVRSPRSSLTEADVQKFIAEQVAPFKRLRRVTFIKTVPKSASGKILRRELIDRVRAKL

>CHS1
MAAKIEEIRQAQRAQGPATVLAIGTATPSNVVYQADYPDYYFRITNSEHLTELKEKFKRMCDKSMIRKRYMHVNEEILQ
ENPNMCAYMAPSLDARQDIVVVEVPKLGKEAAVKAIKEWGQPKSRITHLVFCTTSGVDMPGADYQLTKLLGLRPSVK
RLMMYQQGCFAGGTVLRLAKDLAENSRGARVLVVCSEITAVTFRGPSDTHLDSLVGQALFGDGAAAIIVGADPDPSAE
RPLYQLVSAAQTILPDSHGAIDGHLREVGLTFHLLKDVPGLISKNIEKSLVEAFDPLGISDWNSIFWIAHPGGPAILDQVE
EKLGLKQEKLRATREVLKEYGNMSSACVLFILDEMRKTSAEEGMATTGEGLELGVLFGFGPGLTVETVVLRSLPVPSRC
V

>CHS2
MVGASLEAIRQAQRADGPAAILAIGTATPPNAIEQSTYPDYYFRITNSEHKVELKEKFKRMCEKSMIKKRYMYLTEEILK
ENPNMCAYMAPSLDARQDMVVVEVPKMGKEAAARAIKEWGQPKSRITHLVFCTTSGVDMPGADYQLTKLLGLRPSV
KRLMMYQQGCFAGGTVLRLAKDLAENNRGARVLVVCSEVTAVTFRGPSDTHLDSLVGQALFGDGAAAVIVGADPQE
GVERPLFELVSAAQTILPDSHGAIDGHLREVGLTFHLLKDVPGLISKNIEKSLVEVFEPLGISDWNSLFWIAHPGGPAILD
QVEEKLGLKQEKLRATRAVLRDYGNMSSACMLFILDEIRMKSAEEGRGTTGEGLEWGVLFGFGPGLTVETVVLHSVAT

>CHI
MSPATREEMVAMGLPAVSDVQVEAFVFPPTVRPPGSSKTLFLGGAGARGLEIEGRFIKFTAIGVYLEAAAVPSLAAKWK
GKSPDELAGSVGFFRDVVTGGPYEKFTRVTMILPLTGQQYSEKVAENCVGHWKAIGIYTDAEAEAVEKFKVAFKEETF
PPGSSILFTQSPSGKLTIGFSKDGAVPEAGNAVIENRAMSEAVLESIIGQHGVSPEAQRSLASRVSQLLGEYNPVENGQSE
QVQKKEEPEVVQKDRSVLPEKCNRADDGQAEEAKKD

>F3H
MAPAATLPFLPTAAEEKTLRPSFIRDEDERPKVPYNQFSNDIPVISLAGIDDEGDSSRRVELCRKIVAACEEWGIFQVVDH
GVDAGLIADMTRLAREFFALPPEEKLRFDMSGGKKGGFIVSSHLQGEAVQDWREIVTYFSYPLRTRDYSRWPDKPEGW
RAAVEAYSETVMRLACTLLGVLSEAMGLEREALTKACVDMDQKVVVNFYPKCPQPDLTLGLKRHTDPGTITLLLQDQ
VGGLQATKDGGKTWITVQPVEGAFVVNLGDHGHFLSNGRFRNADHQAVVNSNFCRLSIATFQNPAPDAIVYPLAIREG
EKPVLDEPITFTEMYRRKMSRDLELAKLKKQAKLEEQEALDKAQEAIKHKGMDEILA

>F3’H1
MPTLFLLVLSTVLVGAILHRLLSSTSSRSHCPHLPLPPGPKGWPILGNLPQLGAKPHQTLAALSHAYGPLLHLRFGRVDV
VVASAASAAAAFLKTHDSNFCCRPPNSGAEHVAYNYQDMVFAPYGPRWRVLRKLCSVHLVSAKALDDFRHVREEEA
AALARALLARAGERLGGGGGGQMEVGEEVNVCVMNVLARAVMGRRVFGGGLGREAEEFKEMVVELMRLAGVFNV
GDFVPGLGWLDPQGVVARMKRLHRRYDVMLDKIIAEHAASRGEGNGGDLLSVMVGLRGQAVDADGGTISDVAIKAL
LLDLFTAGTDTTASTVEWALAELIRHRRVLEAAQREIDSVVGRARLVADADLASLPFLQAVVKETFRLHPSTPLSLPRM
ASESCEVAGYHVPKGATLLVNVWAIARDPAAWPRPLEFRPERFLPGGEHAHVDVRGTDFEVIPFGAGRRICAGMNLGL
RMVQFLTATLVHAFDWALPAGQAPENLDMEEAYGLTLQRAIPLKARPIPRLNREAYNAHC

>F3’H2
MASDLLLQLPTIAVASLLLLFLVPKLLGLLFSKPGSRLPLPPGPRGWPVLGNLPQLGPKPHQTLCAMSRAYGPLFHLRLG
SVHVIVAASPAMASLFLKTHDANFCNRPPNTGAMYMAYGYQDLVWAPYGPLWRMLRKICTHHLFSPKALDDSAAVR
REEVAVLAGSLAAAGRAGGVVGVGELLTVCTANILTRAMLGRRLFVAGGAKSDEEARQFKELTVELNTLAGVFMVGD
FVPALRWLNSIGDTVARMKKVGERYDEFLDGIIDQHMASERPQGGHHQEDILRVLMGLKEDAGGGEGVHLTDTVIKA
LLLDLFNAGTDTSSSTVEWTLAELVRHPDILEAARRELDSVVGQGRLVAESDLPRCPLVQAVVKETLRLHPPTPLSLPHI
ASEASDVAGYHVPKGATLLVNVWAIGRDPSVWADPLEFRPARFLPGGQHAHVDVRGNHFEVIPFGAGRRICAGMSLA
LRVIQLITATLIHGFDWAMPEGRAPEKLDMEEAYGLTLQRAVPLRLRPIPRLPVHVYGAAA

>DFR1
MVATKGPLVVTGAAGYIGSWLVMRLLQRGYAVRATVRDPTNLKKVKPLLDLPGAKELLTIWKADVAEEGSFDEVVD
GCVGVFHVATPMDFQSEDPENEVIKPTVDGMLGVLSSCKKAGTVQRVVFTSSAGTVNVEEHQKPEYDESSWSDLDFIW
RVKMTGWMYFVSKTLAEKAAWAFAEENGIHLVSIIPTLVVGPFISSAMPPSLITALALITGNKPHYSIIKQIQLVHLDDLC
NAHIFLFEHSEASGRYICSSHDATIFELAEMIKNRYPEYNVPTEFEGIDNTIQHVRFSSKKLEDLGFEYQYSMEEMFDMAI
QCCREKKLIQLKTEKLCDNEEICEAENKPMRC

>DFR3
MIDKGTVCVTGAAGYVGSWLIMRLLEQGYTVRATVRDPSNMLKVRHLLDLPDAAKRLTLWKADLVDEGSFDEPIQGC
VGVFHVATPMDFESKDPENEVIKPTIEGMLSVLRSCAKTSSVRRVVFTSSAGTVCIQEGRMSLYDERSWSDVDFCRAKK
MTGWMYFVSKTLAEKAAWEFAEKNSIDFISIIPTLVNGPFIMPTMPPSMLSALALITRNEPHYSILNPVQFVHLDDLCNA
HIFLFELPEAKGRYICSSHDVTITSLAKMLRERYPEFDVPTEFGDMQVFDIISFSSKKLLDLGFEFKYSLEDMFDGAIQSCR
EKGLLPPATKEAPAVGK-

>ANS
MATTEVLRAESAPAPPLAPAPPRVESLASSGIEAVPPEYVRPEEERASLTDALEAASKVDEGPQIPTVDMAGFSSGDEAA
RRACVEALLKAASEWGVMHIVNHGIPLDLIRRMQAAGEAFFALPIEEKERYANDQSSGNIQGYGSKLANNASGQLEWQ
DYFFHLIFPEDKANFSIWPKQPANYTEETREFGRQLRVVVSKMLAMLSLGLGLEEGRLDKEVGGMEDLLMQMKINYYP
RCPQPDLAVGVEAHTDVSALSFILHNMVPGLQVYYGGQWVTARCVPDSIIKHVGDVVEILSNGLYKSILHRGLVNKEK

VRISWAVFCEPPRDNILLKPLPELPGPAVFPPRTFSQHIQHKLFRKTRGDFTTPN

>UFGT1
MDRRKVTVVFVPLPFIGHLVPAVQMAKQLLGGRRRHTFSVTVLTLRPLVPFGEMAAYMDSMASSGLDMHFEELPSPEG
DLVTLGNQSPEAIVSLYVERYRPCVRDAVARLSLGVDRPVGAMMMDLFCTALIDVATELGIRPYIYFASSASMLGFML
YTPTLDVLVPSEVEEIGHPIDIPGLQSPVPPLAMPTPMMHKKDSGYTWFLYHARRFREVQGIIVNTFSELEPVAVSAHEE
GLFLRDHSTAPTVYTVGPNIALEFRAGPKHECLGWLDGQPPTSVVFLCFGSWGRFAGAQVKETATGLERSGHRFLWVL
RSPPKHAHNLPTDTVNLREVLPEGFLERTAGRGLVWPSWAPQVDVLAHPSIGGFVTHCGWNSCLESLWFGVPMLAWP
LYAEQHFNAFQMAGDLGVALQLKVDRKNGNFVAAEELERGVRCLMEEGFCERGRKVRARAKEMKTASRRALEDGGS
SLESMERLADELVGVVAGVGEGS

>UFGT2
MGKDSGSGAGDGRLHVAMFPFLAFGHIVPLAQLSKKLAAQGARVSFLSAPANIPRIASLLSPSPVDVLALELPHVEGLPP
EVQSTAEATPATAELLTKAVDLTRPQIQSLLAELRPHAIFHDFAQPWLPSVAHPLGIKTVFFSVFAAVSSAFLTVPSRRPY
PGVSPSVEELRSFDGRPCVFDRDVSCMSASSAIAIKTCREMEAPYIDYVEAQYGKPVLLAGPVVPEALEGELEAKWASW
LESCPAKGSVVFCSFGSETVLTDEGVRELALGLELTGRPFFLVLNFPKGGGASENAAETLRKKLPEGFEEKVRGKGMVH
SGWVQQRHILAHPSVGCFLCHAGLSSVVEGLISGCQLVMLPQRGDQFVNARIFAGDLRAGVEVRRRDEDGGFTRVDVK
AAVRMVMAGEGEQEVPLRTVRESNRRWQEFLLDPEVHRRFTAAFVDKLKEMSGCR

>UFGT3
MASSDSAGRQLQAHVAVFAFPFGAHAAALHALARGLAAAAPAVYFSFFGSAQSNASLPHGDGGPPNLTLYDVSDGLP
AGFAMDPRNPEEMIELFMRVTPGNFREGVEKAVRARGGARISCVVSDAFLWFAADLAGEMAVGVPWVPVRTGGPRG
LSAHIYTDDLRRTIGVGEEAMPHKADEDLGFLPGMSGVRVRDLPEGVVFGNLSSVFARLLHRMGQELPRAAVVAINTF
EGLDPAITDDLRTKLKACLLVGPLNLLCPPQPPPEAECDTSGCLRWLDRQAHGVVAYISFGTQLSPPPRELAELAEELEA
SGAPFLWSLKEKLRAYLPAGFLERTRGQGLVVGWAPQQRVLGHPAVGAFVTHCGWNSVVESVAGGVPLVCRPILGDH
RLNARSVSTLWRVGVEVEGGEMTRDRVAAALGAVLGSEQGKKMKQRICVLGEAARGVARPGGSSAESLRALVKIVSG
V

>AsMYB1

MVKKGEKGGAAAARLAKDKMKVNRGAWTAEEDEKLADFVRAHGDRKWRTLPAKAGLNRCGKSCRLRWLNYLRPG
IKRGNISEQEEDMIIRLHNLIGNRWSLIAGRLPGRTDNEIKNYWNSHLSKKSLTISDLNDKLNGRFETAGTSGSNSDAENA
PISDDLPMVLAGWYEDQSQACQDAQQPAEPWISGEVALDMDELFNFPTLFDFDLRDSGSCGEQDGFLVTSCEDLWTTV
QTAVRGDSSFDNSQLQQGLSDLDELGNLMDDTDYVICPP

>AsMYB2

MLRILRICIRDGQVIPPVYKSKDTAEVAARSVELATMAGAHVHHFTGSGDIRKGAWAPEEDNLLRDCIKKYGEGKWHL
VPDRAGLRRCRKSCRLRWLNYLKPNIKRGKFQEDEVDLILRLHHLLGNRWSLIAGRIPGRTANDIKNYWNACLSKGKA
RAQGEEKLLDQRVVTLNSNSTNPHNPMHCCSYCATFPSATKPTFSTTVIKAQPRNLSKRPHCLPEFNKSSKTSLRETTSN
TYERPVEEDNDSWWKSLFEEETVQEQEPEKQPLEGFINSVHGGTVTGEASAGFCGEGIEVPKGGEERVGSVSWEDLLLD
PSLMRLL

>AsbHLH1

MGALQGGVDGDGCSSGANAPTLQQMLQSAVQSLQWTYSLFWQLCPQQGALVWAEGYYNGAIKTRKVVQPVEATAE
EACLQRSQQLRELYESLSMGETSQQPARRPCAALSPEDLTESEWFYLMCISFFFPPGVGLPGKVFERQQPVWLSGANEV
DSKVFSRAIIAKGAQIQTVVCIPLADGVVEIGTTDKVDEDPALMQHVRSFFTGHQNYPAQTTTKPALSEHSTSNPVPSAP
RQLFRSPSLPAMPPAATDSYRYADEEVEEDDDDDDDDDAGAESDSDSEADARNGGSAEGGGVLRPYAVATAGAAAPPE
AEPSELMQMEMSEEIRLGSPDDCSNNLDADLQMLAVCRTASGQSGGQAGAGPACQAWPLLHDDATNSCLLPSSGATA
TQTMSQEDAHYSQTVSTIMQHNSSRWVDSCSSGYLEHPRQSAFSSWSGRGEHFPAASAGASQWLLKHALRSVPYLHG
KYRGGESSPKQLRDAEGGQRFRKGGALQDELSASHVLAERRRREKLNERFIVLRSLVPCVTKMDKASILGDTIDYLKQL
LRRIQEMETQIKLMESDRRARPVEASKPNHKELSTQTHSSSPPVDAIPHLINDKVRVSGSNKKKPRVLGGGSGPAKAKA
MEDTSVQVSIIEADALLELQCPNRDGLLLKIMQAVHELGLETTAIQSSSADGIFVAEIRAKVRENIHGKRASIMEVKRVL
HLLFSQSLC
